# Metabolomic profiling of VOC-driven interactions between *Priestia megaterium* and *Bacillus licheniformis* in a simulated rhizosphere using split petri dishes

**DOI:** 10.1007/s00203-025-04426-9

**Published:** 2025-08-12

**Authors:** Kamogelo Mmotla, Farhahna Allie, Thendo Mafuna, Manamele D. Mashabela, Msizi I. Mhlongo

**Affiliations:** 1 Department of Biochemistry, Faculty of Science, University of Johannesburg, Auckland Park, 2006 South Africa; 2 Research Centre for Plant Metabolomics, Faculty of Science, University of Johannesburg, Auckland Park, 2006 South Africa

**Keywords:** Rhizosphere, Bacterial communication, Volatile organic compounds, Plant-growth promoting rhizobacteria, Metabolomics

## Abstract

Plant growth-promoting rhizobacteria (PGPR) are bacteria known to enhance plant growth via nitrogen fixation, nutrient solubilization, and phytohormone production. Within the rhizosphere, these bacteria engage in complex intra- and interspecies communication, often mediated by volatile organic compounds (VOCs). VOCs influence microbial behavior, metabolism, and stress responses, yet their specific metabolic impacts remain underexplored. This study applied untargeted metabolomics to investigate VOC-mediated interactions between PGPR strains *Priestia megaterium* and *Bacillus licheniformis*. Using a split petri dish co-cultivation system, we assessed time-dependent changes (days 3, 6, 9) in endo- and exo-metabolomes. Phenotypically, *B. licheniformis* displayed filamentous growth, emerging by day 6 in co-culture but only by day 9 in monoculture, suggesting accelerated morphological adaptation via VOC signaling. Metabolic profiling and multivariate analysis further revealed significant metabolic shifts under co-cultivation, highlighting the strong influence of VOCs on microbial metabolism. In co-culture, *P. megaterium* showed increased secretion of amino acids (e.g. proline, valine) nucleobases (e.g. thymine) and secondary metabolites (macrolactins, bacilliskamide A, oxydifficidin), suggesting VOC-driven activation. In contrast, *B. licheniformis* downregulated secondary metabolite secretion, indicating a trade-off favoring intracellular metabolite retention. Key adaptive response involved metabolic routes related to amino acid use and nitrogen recycling, including pathways for lysine and arginine breakdown, which support energy generation and cellular protection. These findings reveal that VOC-mediated interactions trigger species-specific metabolic reprogramming, influencing microbial dynamics and potentially enhancing plant-microbe associations, with implications for sustainable agriculture.

**Supplementary Information:**

The online version contains supplementary material available at 10.1007/s00203-025-04426-9.

## Introduction

The rhizosphere harbors a vast array of microbes, making it the most biologically diverse region on Earth (Berendsen et al. 2012). This zone serves as the interface for microbial interactions and plant roots. The collective community of microorganisms residing in the vicinity of the root zone is referred to as the rhizosphere microbiome (Ali et al. 2017). Plant Growth-Promoting Rhizobacteria (PGPR), a subset of these microbes, play crucial roles in enhancing plant growth through various mechanisms such as nitrogen fixation, nutrient solubilisation and phytohormone production (Mhlongo et al. 2020; Fierer et al. 2021; Mashabela et al. 2022). Two commonly studied PGPR species, *Priestia megaterium* and *Bacillus licheniformis*, exemplify microbes that contribute significantly to plant health and soil fertility (Akhtar et al. 2020; Biedendieck et al. 2021). To maintain population stability and optimise their role as key players in terrestrial biogeochemical cycles, these diverse soil microorganisms form intricate yet stable predictable communities. They engage in ecological interactions not only with other soil microbes but also among themselves, creating a complex consortium in the edaphic environment. Importantly, microbial interactions within these consortia-mediated through inter- or intra-species cell-to-cell communication are highly diverse and sophisticated.

These interactions within the soil microbiome, which encompass mutualism, parasitism, predation, amensalism, and competition can impact the components in positive, negative or neutral way (Faust and Raes 2012). However, for microbes to engage in these types of interactions they use various mechanisms including quorum sensing, signaling molecules like acyl homoserine lactones (AHLs), cyclic dipeptides, secondary metabolites, siderophores, biofilm formation, nutritional interdependencies, antibiotics, lipopeptides, among others (Bhattacharyya and Jha 2011; Hassani et al. 2018; Jamil et al. 2022). Other common compounds used for interaction are volatile organic compounds (VOCs), which serve as effective information carriers, enabling short and long-range signaling, as well as inter and intra-organismal communication both above and below ground (Fincheira and Quiroz 2018; Menezes et al. 2021) VOCs are low-molecular-weight lipophilic biochemicals (< 500 Da) generated by a variety of bacterial and fungal species through distinctive metabolic processes that are genotype specific (Kanchiswamy et al. 2015; Fincheira and Quiroz 2018). For instance, four volatiles produced by *Collimonas pratensis* and *Serratia plymuthica*- specifically methanthiosulfonate, S-methyl thioacetate, dimethyldisulfide and benzonitrile, were individually tested for their impact on *Pseudomonas fluorescens*. Benzonitrile and dimethyldisulfide promoted the growth of *P. fluorescens*, while methanthiosulfonate and S-methyl thioacetate showed no effect on the growth of *P. fluorescens* (Garbeva et al. 2014). Other VOCs such as alkanes, ketones, alkenes, terpenoids and sulphurs are among the VOCs of bacterial origin. These compounds influence microbial behaviour, such as virulence, stress resistance and biofilm formation (Soto et al. 2021) Some are very effective in preventing bacterial growth or fungal growth, as reported in previous studies that *Pseudomonas* and *Bacillus* strains with growth-promoting capabilities can emit VOCs with antimicrobial activity (Raza et al. 2016; Rajer et al. 2017), VOCs thus play a significant role in microbe-microbe communication (Audrain et al. 2015). Additionally, microbe-microbe interactions can cause metabolic changes and reprogramming in neighbouring microbial species, which leads to either positive or negative changes in behaviour. Positive interactions include the perception of signals from one bacterial strain to induce synergistic intercommunication for increased mutual interactions, while negative interactions may cause the demise of one microbe for the survival of another.

The composition of VOCs from rhizobacteria is influenced by factors such as genomic makeup, metabolic capacity, developmental stage, and nutrient availability in the surroundings (Groenhagen et al. 2013). Variations in VOCs profiles can differentiate between strains of the same rhizobacterial species, underscoring the specific nature of their production. While the role of mVOCs in species interaction is well documented (Mmotla et al. 2025), their specific effects on the interacting organisms have not been fully elucidated. Hence in this study we investigate the changes induced by mVOCs of neighboring bacteria both internally and externally, using metabolomics. Microbial metabolomics, especially the examination of metabolic changes in bacteria, has attracted considerable research attention (Kumar et al. 2017; Mohd Kamal et al. 2022). At the cellular level, metabolomics offers a comprehensive snapshot and detailed characterisation of important metabolic changes associated with the phenotype. These changes can be classified by the metabolite’s origin, either endogenous (originating within the cell) or exogenous (originating outside the cell), and either function, categorised as primary or secondary metabolites (Horak et al. 2019). Primary metabolites are involved in pathways related to the synthesis of (anabolic activity) and breakdown (catabolic activity) of these compounds. In contrast, secondary metabolites are associated with growth rates and stress responses (Pinu et al. 2017). Metabolomics proves to be valuable in studying PGPR-PGPR interactions (Luzzatto-Knaan et al. 2019; Menezes et al. 2021). By analysing the complete set of metabolites present in these interactions, a though understanding can be gained into how these beneficial bacteria interact and cooperate to enhance plant growth. Furthermore, the integration of metabolomics and chemometrics tools enables a thorough categorisation, distinguishing both similarities and differences (Zhang and Powers 2012; Broughton-Neiswanger et al. 2020) and classifying metabolic patterns or profiles induced by neighboring bacteria via VOCs. This approach identifies biomarkers responsible for observed phenotypes, enhancing our understanding of the intricate interactions between PGPR in the rhizosphere. Such insights are crucial for developing sustainable farming practices that harness these microbial interactions to optimise crop productivity while reliance on synthetic chemicals.

## Materials and methods

### Bacterial isolation

The PGPR used in this study were *P. megaterium* and *B. licheniformis*. In brief, *B. megaterium* was obtained from (University of Johannesburg, South Africa), it was maintained in − 80% glycerol at − 80 °C. Meanwhile, *B. licheniformis* was isolated from microbial inoculant (Efficient Microbes Pro-soil, Westville KZN, S.A) and maintained in 80% glycerol stocks at − 80°.

### Preparation of petri dishes using M9 media agar

To prepare the petri dishes, 15 mL of M9 media agar composed of 2.56% (w/v) Na_2_HPO_4_ (Merck (Pty) Ltd, S.A), 0.6% (w/v) KH_2_PO_4_ (Merck KGaA, Germany), 0.1% (w/v) NaCl_2_ (RLS chemicals, S.A), 0.2% (w/v) NH_4_Cl_2_ (Merck KGaA, Germany), 0.05% (w/v) MgSO_4_ (Merck KGaA, Germany), 0.002% (w/v) CaCl_2_ (Sigma-adrich, Germany), 0.1% (w/v) Glucose (Acechem, S.A), 0.2% (w/v) malic acid (Merck KGaA, Italy) and 1.5% (w/v) of agar (Sigma-adrich, Germany)) was poured and evenly distributed in each petri dish. Ensuring even distribution is crucial for obtaining uniform microbial growth and accurate results in subsequent experiments. The petri dishes were then allowed to cool and solidify completely at room temperature, ensuring a smooth, level surface optimal for inoculating with bacterial cultures.

### Bacterial mono-cultures and co-cultures

Monocultured strains of *P. megaterium* (PM) and *B. licheniformis* (BL) were grown on M9 medium and served as a control. Co-cultures of *P. megaterium* with *B. licheniformis* (PM-BL) and *B. licheniformis* with *P. megaterium* (BL-PM) were also grown on M9 medium using as split (two compartments) petri dish and all the plates were incubated at 32 °C with thermal precision of ± 2 °C. The study was conducted in two independent setups, each with three replicates for monocultured bacterial strains and three replicates for co-cultured bacterial strains.

### Sample harvesting

Harvesting was carried out in three-day intervals (days 3, 6, and 9). For endo-metabolite harvesting, bacterial colonies were scraped from the agar using an inoculation loop and transferred into a 2 mL centrifuge tube. Following the complete removal of bacterial cells from agar plates, the agar was cut into slices and transferred into 50 ml tubes (for exo-metabolites), and the samples were stored at −80 °C until extraction.

### Metabolite extraction

#### Endo-metabolite extraction

Bacterial colonies stored in 2 mL centrifuge tubes were freeze-dried and the dried biomass (0.5 g) was standardised by accurately weighing each sample. An appropriate volume (1 mL) of extraction solvent (50% methanol and 0.1% Formic acid) was then added proportionally to the sample weight to ensure consistency in metabolite recovery across all samples. The samples were then sonicated at 55% power for approximately 30 s using a sonicator, followed by centrifugation at 3200 rpm for 15 min at 4 °C, the supernatant was transferred to new 2 mL tubes, the samples were then dried at 55 °C using a dry bath. All samples were reconstituted using extraction solvent (50% Methanol and 0.1% Formic acid) and then filtered through 0.22 μm nylon syringe filters into 2 mL vials for analysis.

#### Exo-metabolite extraction

Metabolites were extracted according to Tyc et al. (2017a, b) with minor modifications. The frozen media samples were subsequently freeze-dried for 72 h to ensure complete dehydration. The freeze-dried samples were then weighed, and their masses were adjusted as needed to achieve uniformity across the samples. An extraction solvent consisting of 50% methanol and 0.1% Formic Acid was added to each 50 mL tube containing freeze-dried media. The samples were sonicated at 55% power for approximately 30 s using a probe sonicator, followed by centrifugation at 4700 rpm for 15 min at 4 °C, the supernatant was transferred to new 15 mL tubes, the samples were then dried at 55 °C using a dry bath. All samples were reconstituted using extraction solvent (50% Methanol and 0.1% Formic acid) and then filtered through 0.22 μm nylon syringe filters into 2 mL vials for analysis. Flirted samples were stored at −4 °C until UPLC-MS analysis to preserve metabolite stabilty.

### Metabolomics-based data acquisition, analysis, and interpretation

#### Ultra performance liquid chromatography-mass spectrometry (UPLC-MS) analysis

The extracts were analysed to identify various metabolites using acquity ultra-high performance liquid chromatography coupled with SYNAPT XS quadrupole time-of-flight Mass Spectrometer (r (Waters, Milford, MA, USA). Chromatographic separation was achieved using an ACQUITY™ PREMIER HSS T3 1.8 μm Column (Waters, Milford, MA, USA), 1/pk. Solvent A was water with containing 0.05% formic acid (FA) and 0.05% isopropyl alcohol (IPA), and solvent B was acetonitrile with IPA. The gradient elution protocol was as follows: 100% A to 0.0% B initially at 0 min, 100% A to 0.0% B from 0 to 1 min; 10% A to 90% B from 1 to 15 min; 1.0% A to 99.0% B from 15 to 15.10 min; 1.0% A to 99.0% B from 15.10 to 17 min; 100% A to 0.0% B from 17 to 17.10 min; and 100% A to 0.0% B from 17.10 to 20 min. The injection volume was 2 µl with a flow rate of 0.4mL/min. The instrument scanned mass range of 100–1500 Da. The source parameters were as follows: capillary voltage of + 2800 V, cone voltage of + 30 V, source temperature set at 120 °C, desolvation temperature at 450 °C, desolvation gas flow at 600 L/h, and cone gas flow at 5 0 L/h. Data were collected in centroid mode at an approximate resolution 10,000, with a scan time of 0.1 s to ensure over 10 data points per chromatographic peak. Internal mass calibration was achieved using the Lockspray interface (Waters), with a continuous infusion of leucine-enkenphalin (500 ng/mL) at 15 µL/min. System operation and data acquisition were managed using MassLynx 4.1 software (Waters).

#### Data processing, multivariate data analysis, annotation and biological interpretation

Data processing was carried out in Markerlynx XS (Waters) using the following parameters: a retention time range of 0.64–18.2 min, mass range of 100–1500 Da, mass tolerance of 0.05 Da, and a noise elimination threshold of 10. For multivariate data analysis (MVDA), pre-processed data matrices were exported to MetaboAnalyst 6.0 (Pang et al. 2024). Statistical analyses, including Principal Component Analysis (PCA) and heatmap generation, were conducted to describe the results quantitatively. Additionally, the Variable Importance in Projection (VIP) score plots were utilized to illustrate the separation and distinctive features between mono-cultured and co-cultured organisms. The VIP scores reflect the extent to which each metabolite influences the model’s capacity to distinguish between groups, thereby facilitating the identification of significant differential metabolites.

The identification of key ions influencing metabolite profiles in mono-cultured and co-cultured strains varieties was facilitated by using the freely available MS-DAIL software (version 4.90) (Tsugawa et al. 2020). The raw data files were first converted into the ABF format using Reifycs Analysis Base File Converter (https://www.reifycs.com/AbfConverter/). These converted ABF files were subjected to a series of data processing steps, including peak picking, alignment, deconvolution, and identification, in MS-DAIL software. The parameters applied in MS-DAIL included MS1 and MS2 mass tolerance set at 0.02 Da, respectively, a retention time 0.67-17, MS1 and MS/MS mass range 100–1500, minimum peak height threshold of 1500, a mass slice width of 0.01 Da, and a linear-weighted moving average smoothing method using 3 scans with a peak width of 5 scans.

The process followed Level 2 guidelines established by the Metabolomics Standards Initiative (MSI), ensuring annotations were based on similarities with published data, including spectral patterns and elemental compositions. To further refine identifications, the m/z values of selected compounds were used to derive their empirical formulae, which were then queried against online databases including MassBank Europe (https://massbank.eu/MassBank/) and Pubchem (https://pubchem.ncbi.nlm.nih.gov/) to pinpoint matching compounds. Pathway analysis of the metabolites was conducted using the MetPA tool within MetaboAnalyst 6.0, facilitating the analysis, identification, and visualization of affected pathways. MetPA utilizes high-quality KEGG metabolic pathways as its underlying knowledge base. Employing MetPA for pathway analysis provided a framework for partially mapping the molecular landscape of metabolism under study, enabling the biological interpretation of observed changes in the endo-metabolome.

## Results

### Phenotypic changes in PGPR cultures influenced by VOC exchange overtime

**Fig. 1:**
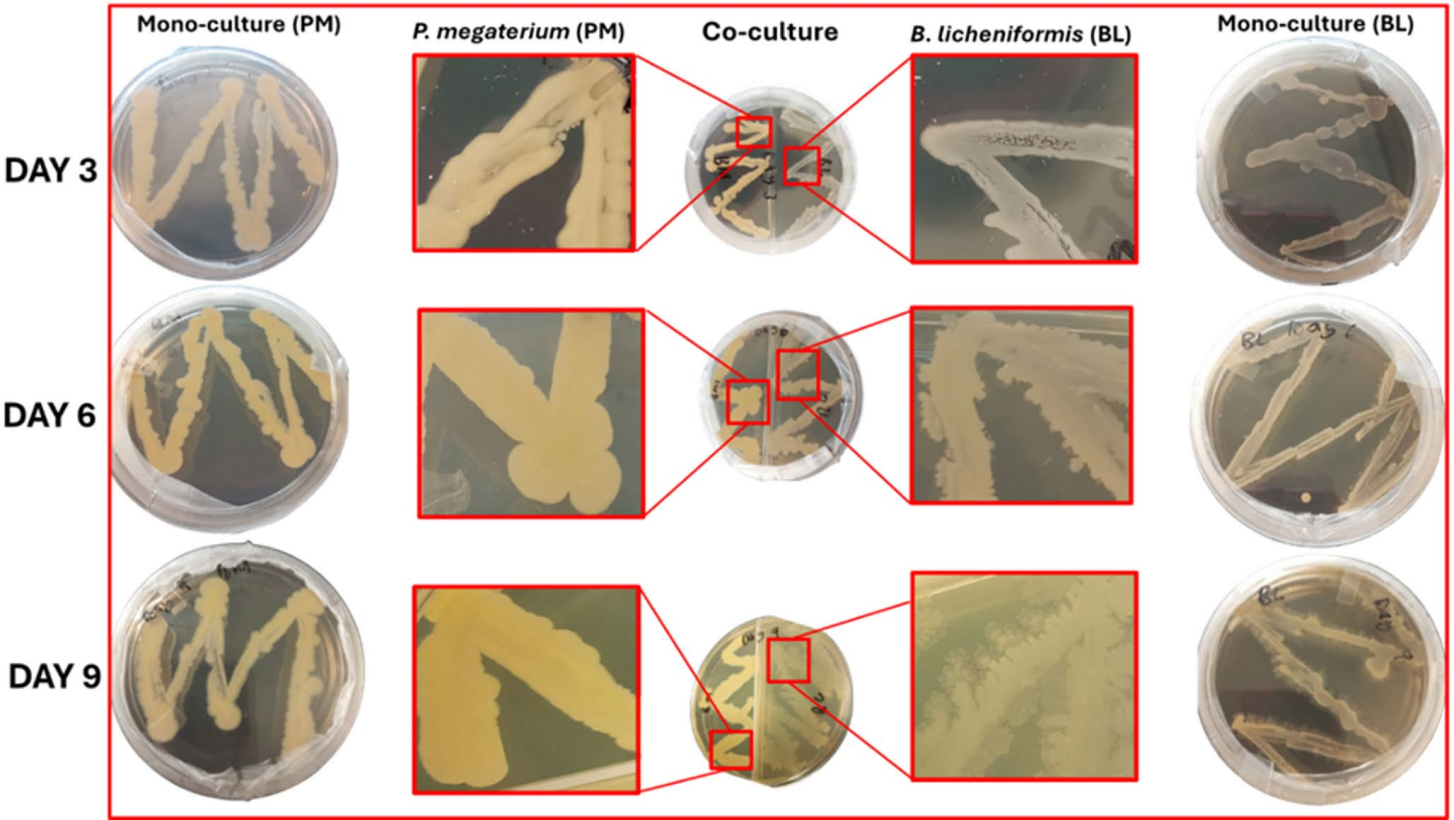
Phenotypic changes observed in *P. megaterium* (PM) and *B. licheniformis* (BL) in response to VOCs over a period of 9 days. The two PGPR were cultured on M9 media, with phenotypic observations recorded at 3-days intervals (days 3, 6 and 9). Monocultured PGPR serves as the control, while the co-cultured PGPR represents experimental groups exposed to each other’s VOCs. The observed phenotypic changes indicate a dynamic response to VOCs, with *B. licheniformis* exhibiting significant alteration in colony morphology, particularly by day 9

Phenotypic changes were observed during sampling. On day 3, no significant phenotypic alterations were observed. However, by day 6, *B. licheniformis* exhibited filamentous strands in monoculture plates, with even more pronounced filamentous development observed in co-cultured plates (Fig. 1), likely driven by exchange of VOCs such as 2,3-butanediol, aldehydes, and esters, which are known to influence bacterial morphology and growth dynamics (Bitas et al. 2013; Augusto et al. 2023; Mmotla et al. 2025). By day 9, the filamentous structures of *B. licheniformis* had become more prominent and particularly in co-cultured with *P. megaterium* (Fig. 1). These findings suggest that VOCs play a significant role in enhancing the filamentous growth of *B. licheniformis*, indicating a possible interaction between the two PGPR mediated by VOCs, that promotes morphological changes. In contrast, *P. megaterium* did not exhibit filamentous growth under standard culture conditions (Fig. 1). The absence of such structures suggests that *P. megaterium*, under the tested conditions, may not undergo similar morphological changes or respond to the same environmental cues that promote filamentation in *B. licheniformis.* This observation is consistent with known strain-and species-specific variability in morphological plasticity and response to environmental stimuli among soil-dwelling bacteria (López and Kolter 2010).

### Co-cultivation of *P. megaterium* and *B. licheniformis* reveals dynamic shifts in their metabolic profiles

**Fig. 2:**
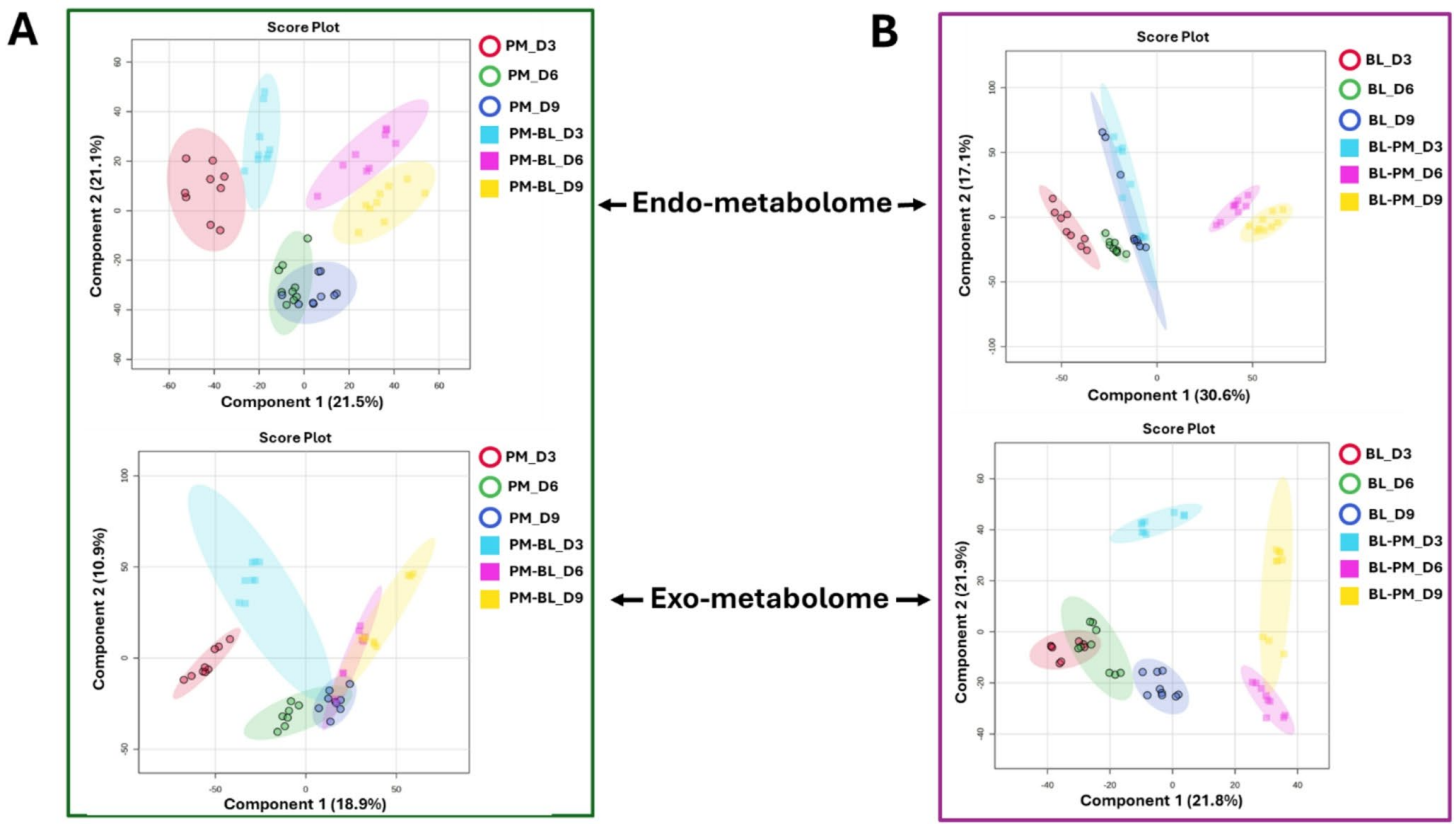
Supervised partial least squares discriminant analysis (PLS-DA) models comparing PGPR monoculture and co-cultured samples. **A** *P. megaterium* and **B** *B. licheniformis*. The score plots illustrate distinct variations in the endo- and exo-metabolome, highlighting the impact of VOCs and potential response mechanisms. The data were median-normalized, log-transformed, and Pareto-scaled in MetaboAnalyst to enhance correlation and predictive accuracy

To assess the dynamics of metabolite perturbation during co-cultivation of the bacterial strains, PLS-DAs were performed. This supervised approach facilitates the visualisation of distinct separation and clustering patterns of both endo and exo-metabolic data across various time points. In *P. megaterium*, the endo-metabolic data exhibited a distinct separation between mono-cultured and co-cultured conditions across time points (Fig. 2A). However, an exception was observed on day 3, where both conditions *P. megaterium* (PM_D3) and *P. megaterium* co-cultured with *B. licheniformis* (PM-BL_D3) clustered together (Fig. 2A), suggesting a temporary convergence in metabolic activity.

In contrast, the exo-metabolic data of *P. megaterium* exhibited clustering between monoculture and co-culture conditions at day 6 and 9, suggesting a period of metabolic convergence (Fig. 2A). This pattern indicates that extracellular metabolite production is strongly influenced by the cultivation method, potentially due to shared environmental factors, metabolic exchanges, or regulatory responses. Similarly, for *B. licheniformis*, the endo-metabolic data clustered at day 3 and 9 (Fig. 2B), suggesting metabolic similarities at these specific time points. However, unlike *P. megaterium*, the exo-metabolic data of *B. licheniformis* displayed a clear separation, highlighting distinct extracellular metabolite production patterns. Despite the components explaining < 50% of the total variance, the observed group separation remain meaningful due to validation metrics supporting the robustness of the model. We conducted cross-validation (Fig. S1.1) which demonstrated consistent classification accuracy across folds, indicating the model’s generalizability.

### Time-dependent metabolic reprogramming reveals the role of primary and secondary metabolite production as a key driver of in inter-specific variations in endo-metabolic profiles

**Fig. 3:**
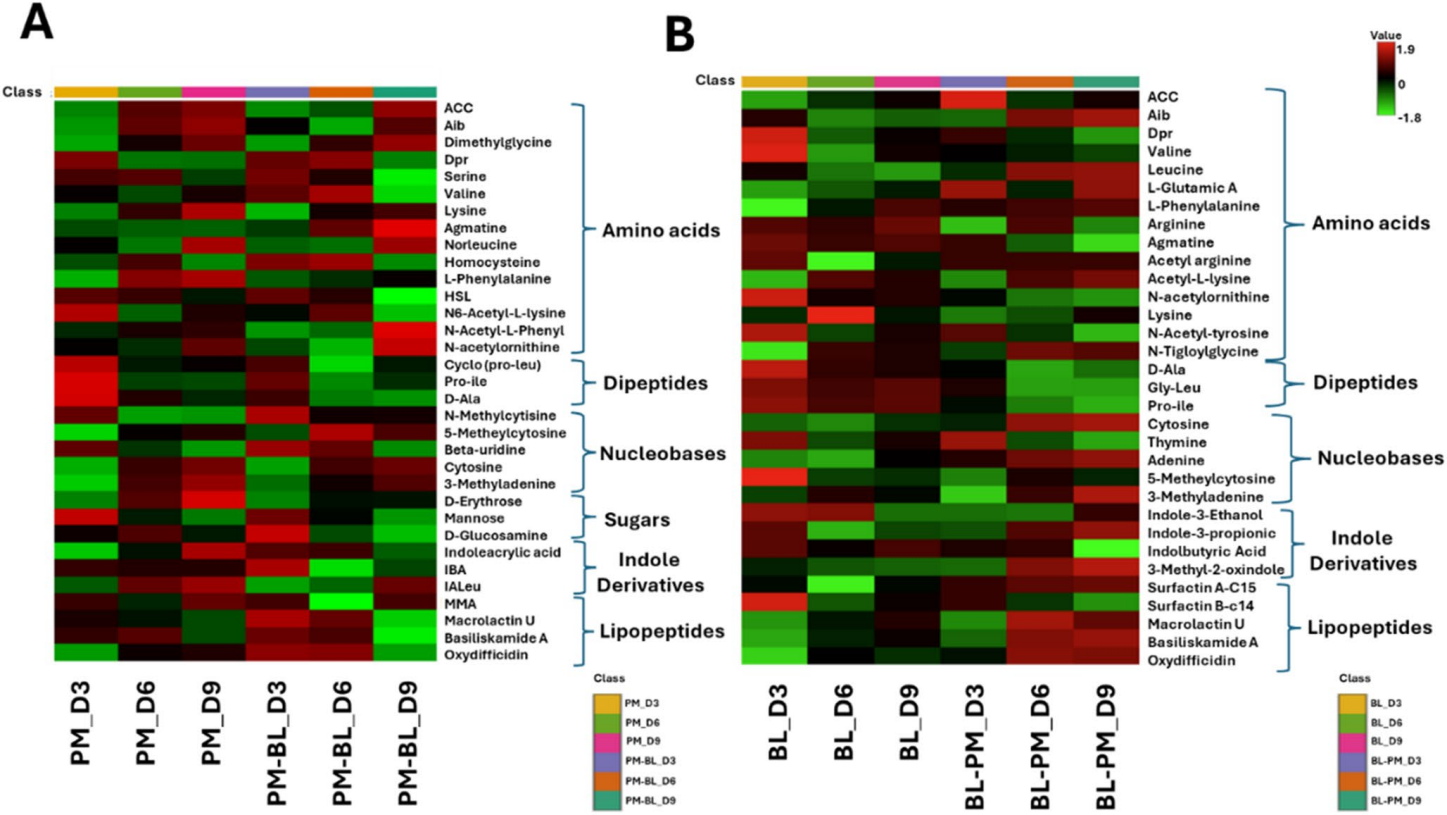
Differential quantitative profiles of annotated endo-metabolites. The interactive heatmaps were generated using the average peak intensities of metabolites (*n* = 9) in MetaboAnalyst. Data were median-normalized, log-transformed, and Pareto-scaled for enhance comparability. **A** *P. megaterium* and **B** *B. licheniformis*. The heatmaps illustrate metabolic perturbations induced by volatile organic compounds (VOCs) during co-cultivation, highlighting changes in metabolite abundance. Metabolite annotations were performed using MS-DIAL and further validated according to Level 2 classification of the Metabolomics Standards Initiative (MSI-2). *ACC* 1-aminocyclopropane-1-carboxylate; *Aib* 2-amino-2-methylpropanoate; *Dpr* 2,3-diaminopropionic acid; *HSL* Homoserine lactone; *Pro-ile* L-Prolyl-l-isoleucine; *D*-*ala* d-alanine-d-alanine; *Gly-Leu* Glycine-Leucine, *IBA* Indole-3-butanoic acid; IALeu = Indole-3-acetyl-L-leucine; MMA = 7-O-Malonyl macrolactin A

The interactive heatmaps were generated using MetaboAnalyst 6.0, to visualise metabolite distribution patterns. These heatmaps revealed variations in metabolite accumulation across primary metabolites, including amino acids, dipeptides, nucleobases, as well as secondary metabolites, such as indoles and lipopeptides. As illustrated in Fig. 3, these differences suggest distinct metabolic adaptations in response to co-cultivation, providing insights into the dynamic shifts in metabolite production. Furthermore a total of eight metabolites with overall VIP > 1 were identified for *B. licheniformis*, and four for *P. megaterium*, indicating their key contributions to class separation in the PLS-DA models (Fig. S1.2).

**Fig. 4:**
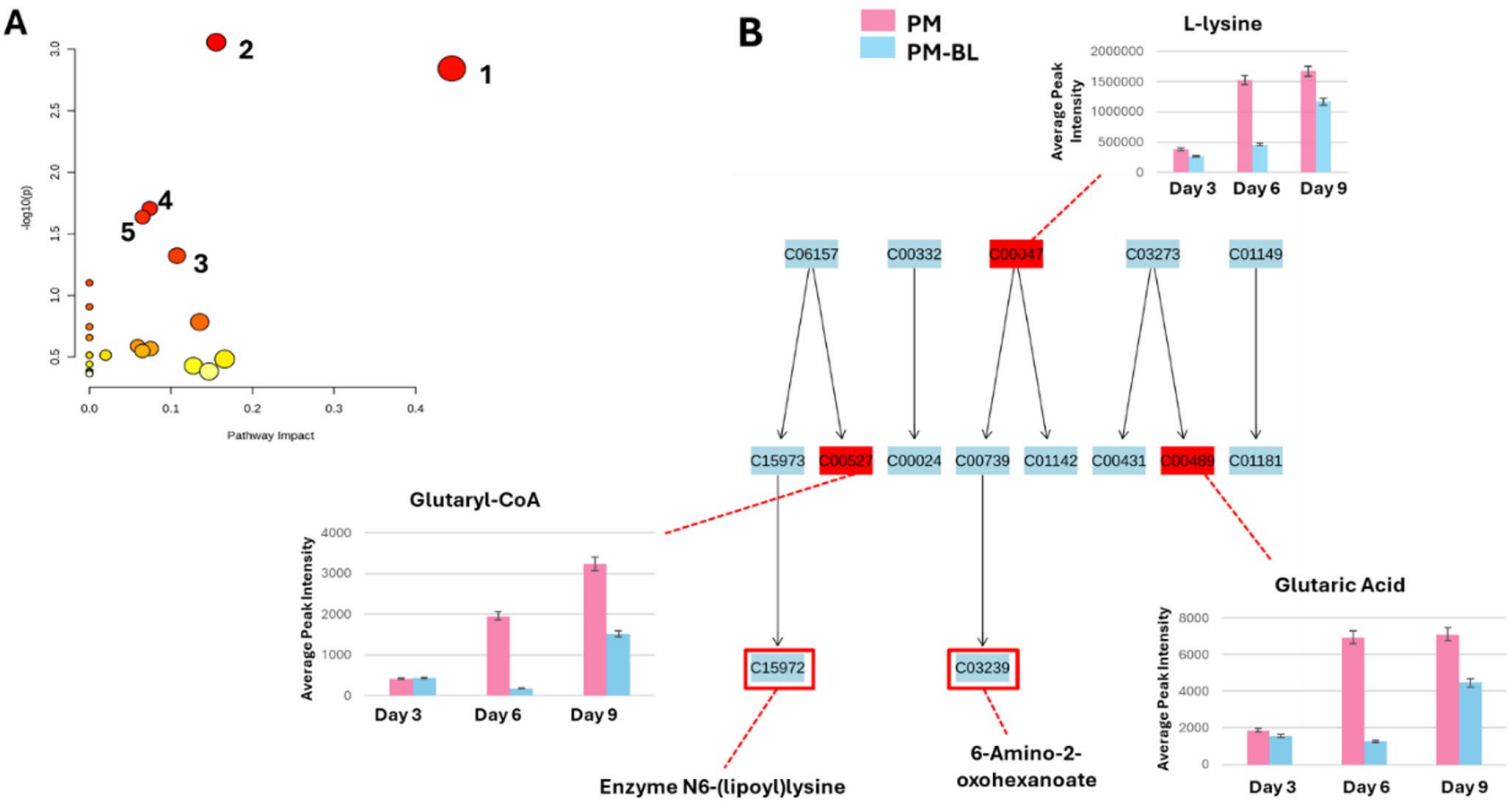
Metabolic pathway analysis and relative quantification of altered metabolites in the lysine degradation pathway. **A** Pathway analysis for *P. megaterium* (PM), highlighting statistically significant metabolic pathways identified based on matched metabolites (1) Lysine degradation (2) D-amino acid metabolism (3) Cystine and methionine metabolism (4) Pyrimidine metabolism (5) Sulfur metabolism. Pathways are ranked according to their impact values (x-axis) from pathway topology analysis and their statistical significance (*p*-value) from enrichment analysis (y-axis). Node color represents the *p*-value (red = highest significance), while node size corresponds to pathway impact, with larger nodes indicating greater influence. **B** Topological representation of the lysine degradation pathway, showing the relative quantification (averaged peak intensities) of mapped metabolites. These panels illustrate metabolic variations during monoculture and co-cultivation from day 3 to day 6. Some of the unannotated end products of the lysine degradation pathway are indicated in red demarcations

By examining time-dependent changes in the heatmap, we aimed to identify distinct metabolic patterns that difference between mono and co-cultured conditions, providing insight into their metabolic profiles. This approach enabled us to assess the dynamics of metabolite accumulation over time, offering a more comprehensive understanding of how VOCs influence metabolic output. In the case of *P. megaterium*, co-cultivation resulted in a noticeable decrease in several metabolite classes including amino acids and lipopeptides, compared to monoculture (Fig. 4 A). Conversely, when *B. licheniformis* was co-cultivated with *P. megaterium*, a marked increase in the production of amino acids, indole derivatives, and lipopetides was observed particularly on day 6 and 9 (Fig. 4B). This suggests that VOCs from *B. licheniformis* could have a stimulatory effect, promoting the synthesis of these metabolites. Furthermore, the interaction between the two strains appears to be time-dependent, with VOCs exerting a cumulative or delayed impact on metabolite production.

### The metabolic pathway alterations in *P. megaterium* and *B. licheniformis* reveal VOC-driven adaptive response

Metabolic pathway analysis (MetPA) was performed using Metaboanalyst 6.0, incorporating matched KEGG IDs of putatively annotated metabolites to identify and visualise the most significantly impacted pathways (*p*-value < 0.05) associated with the observed metabolic reprogramming. Pathways were ranked based on their pathway impact (x-axis). For *P. megaterium*, the most significantly affected metabolic pathways included lysine degradation, D-amino acid metabolism, pyrimidine metabolism, sulfur metabolism, and cysteine and methionine metabolism, as depicted in the topology diagram (Fig. 4A). Notably, the lysine degradation pathway emerged as the most impacted pathway with *p*-value of 0.006 and pathway impact of 0.4. This is particularly relevant as lysine degradation plays a key role in amino acid catabolism and energy production, influencing various biosynthetic processes. Furthermore, time-point analysis revealed that the annotated metabolites, including key end products such as N6-(lipoly) lysine and 6-amino-2-oxohexanoate, were significantly affected during the co-cultivation period (day 3,6 and 9). These metabolites were notably more abundant under monoculture but exhibited a marked decline in co-culture (Fig. 4B). This suggests that VOCs play a critical role in modulating metabolic outputs, potentially influencing nutrients exchange, metabolite synthesis, and overall metabolic dynamics within the system.

**Fig. 5:**
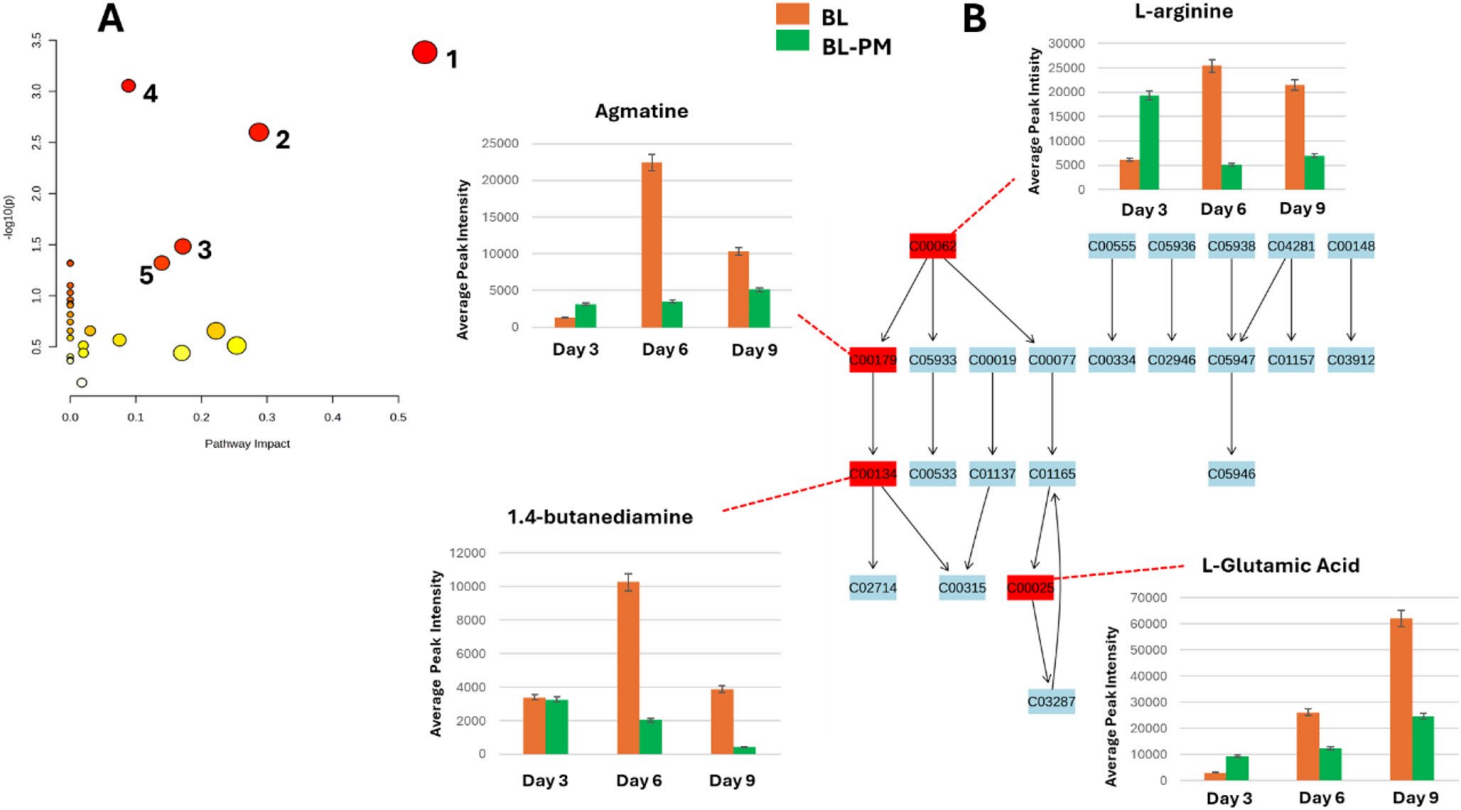
Metabolic pathway analysis and relative quantification of altered metabolites in the arginine and proline pathway. **A **Pathway analysis for *B. licheniformis* (BL), highlighting statistically significant metabolic pathways identified based on matched metabolites (1) Arginine and proline metabolism (2) Arginine metabolism (3) D-amino acid metabolism (4) Glutathione metabolism (5) Pantothenate and CoA biosynthesis. Pathways are ranked according to their impact values (x-axis) from pathway topology analysis and their statistical significance (p-value) from enrichment analysis (y-axis). Node color represents the p-value (red = highest significance), while node size corresponds to pathway impact, with larger nodes indicating greater influence. **B **Topological representation of the arginine and proline pathway, showing the relative quantification (averaged peak intensities) of mapped metabolites. These panels illustrate metabolic variations during monoculture and co-cultivation from day 3 to day 6

In *B. licheniformis*, several metabolic pathways were significantly affected, including arginine and proline metabolism, arginine biosynthesis, D-amino acid metabolism, glutathione metabolism, and pantothenate and CoA biosynthesis (Fig. 5A). Among these, arginine and proline metabolism emerged as the most significantly impacted pathway with a *p*-value of 0.04 and pathway impact of 0.5 (Fig. 5A), suggesting its critical role in the bacterium’s adaptive metabolic response. Key metabolites within this pathway, including agmatine, L-arginine, L-glutamic acid, and 1,4-butanediamine, showed an initial increase on Day 3 but declined on Days 6 and 9, indicating dynamic metabolic shifts over time (Fig. 5B). The perturbation of this pathway suggests a potential shift in nitrogen metabolism, as arginine and proline are key intermediates involved in cellular stress responses, protein biosynthesis and energy metabolism (Scribani Rossi et al. 2022).

### The secretion of diverse primary and secondary metabolites contributes to interspecific differences in exo-metabolic profiles

**Fig. 6:**
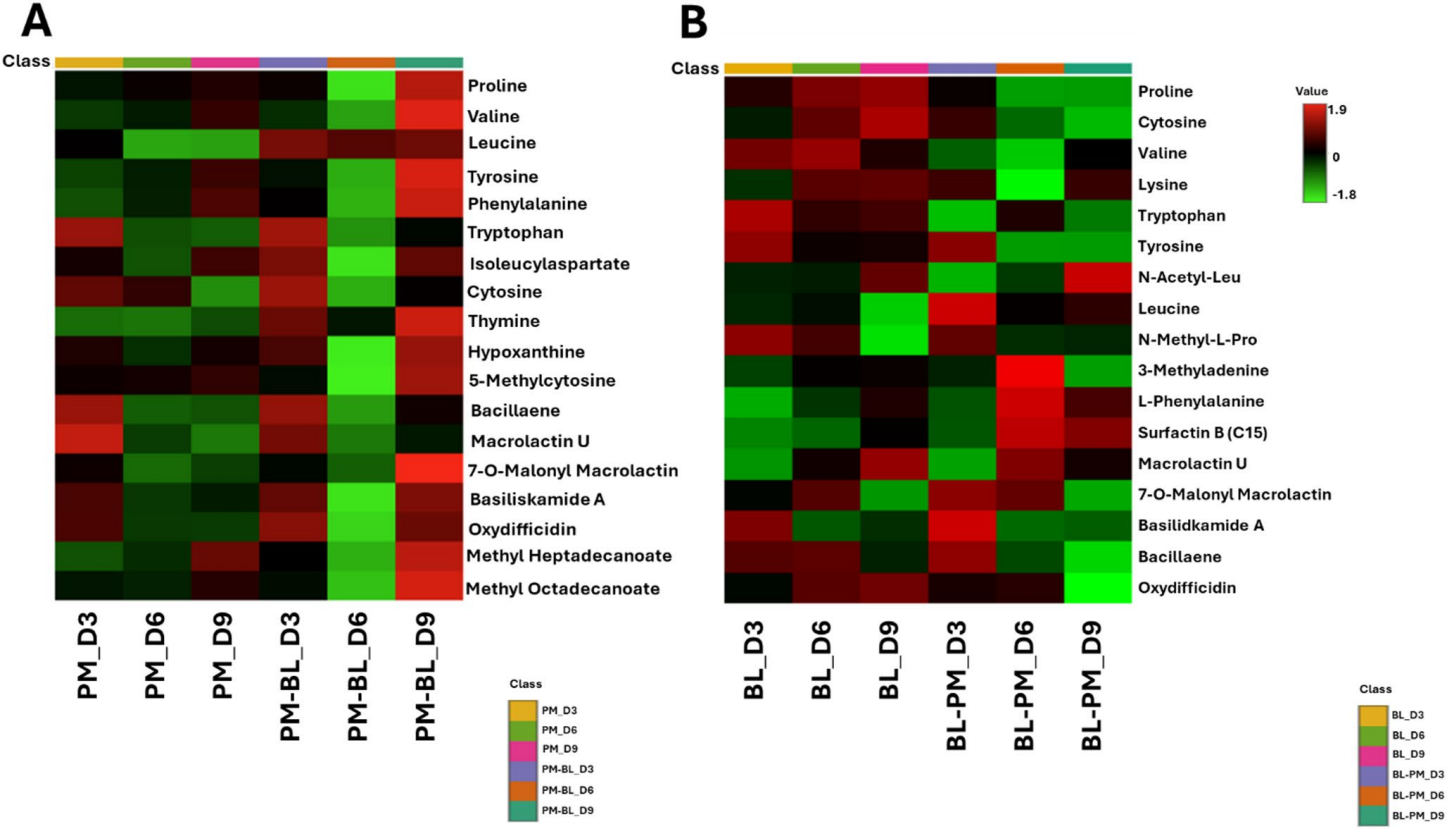
Differential quantitative profiles of annotated exo-metabolites. The interactive heatmaps were generated using the average peak intensities of metabolites in MetaboAnalyst. The data were median-normalized, log-transformed, and Pareto-scaled for enhanced comparability. **A ***P. megaterium* and **B ***B. licheniformis*. These heatmaps display the exo-metabolites secreted by PGPR into the media during monoculture and co-cultivation, highlighting variations in metabolite abundance and classification

A heatmap was generated to analyse the profile of metabolites secreted into the culture media, providing a visual representation of their dynamic changes over time. This approach facilitated the identification of key metabolic shifts, highlighting variations in the composition and abundance of secreted primary and secondary metabolites under mono and co-cultivation. Comparative analysis revealed a distinct pattern of metabolic variation between the two bacterial species. Notably, the secretion of primary metabolites, particularly amino acids, was significantly increased in the co-culture medium of *P. megaterium* compared to its monoculture, suggesting that VOCs from *B. licheniformis* stimulated amino acid secretion (Fig. 6A).

Conversely, in *B. licheniformis* exhibited a noticeable decrease in primary metabolites including amino acids levels in its co-culture media compared to its monoculture control. This decline suggests that VOCs from *P. megaterium* may have influenced *B. licheniformis* by altering its metabolic priorities, potentially suppressing amino acids secretion (Fig. 6B). The production of secondary metabolites, including bacillaene, macrolactins, and bacilliskamide A, were notably elevated in *P. megaterium*, particularly on day 3 and 9 compared to its monoculture. In contrast, *B. licheniformis* exhibited a reduction in secondary metabolite production under co-culture conditions. This decrease suggests that VOCs from *P. megaterium* may have disrupted or downregulated the biosynthetic pathways of *B. licheniformis*, leading to a metabolic shift that prioritised alternative pathways over secondary metabolite production.

### Correlation analysis of endo- and exo-metabolites reveals metabolic interconnections and regulatory patterns in co-cultivated bacteria

**Fig. 7:**
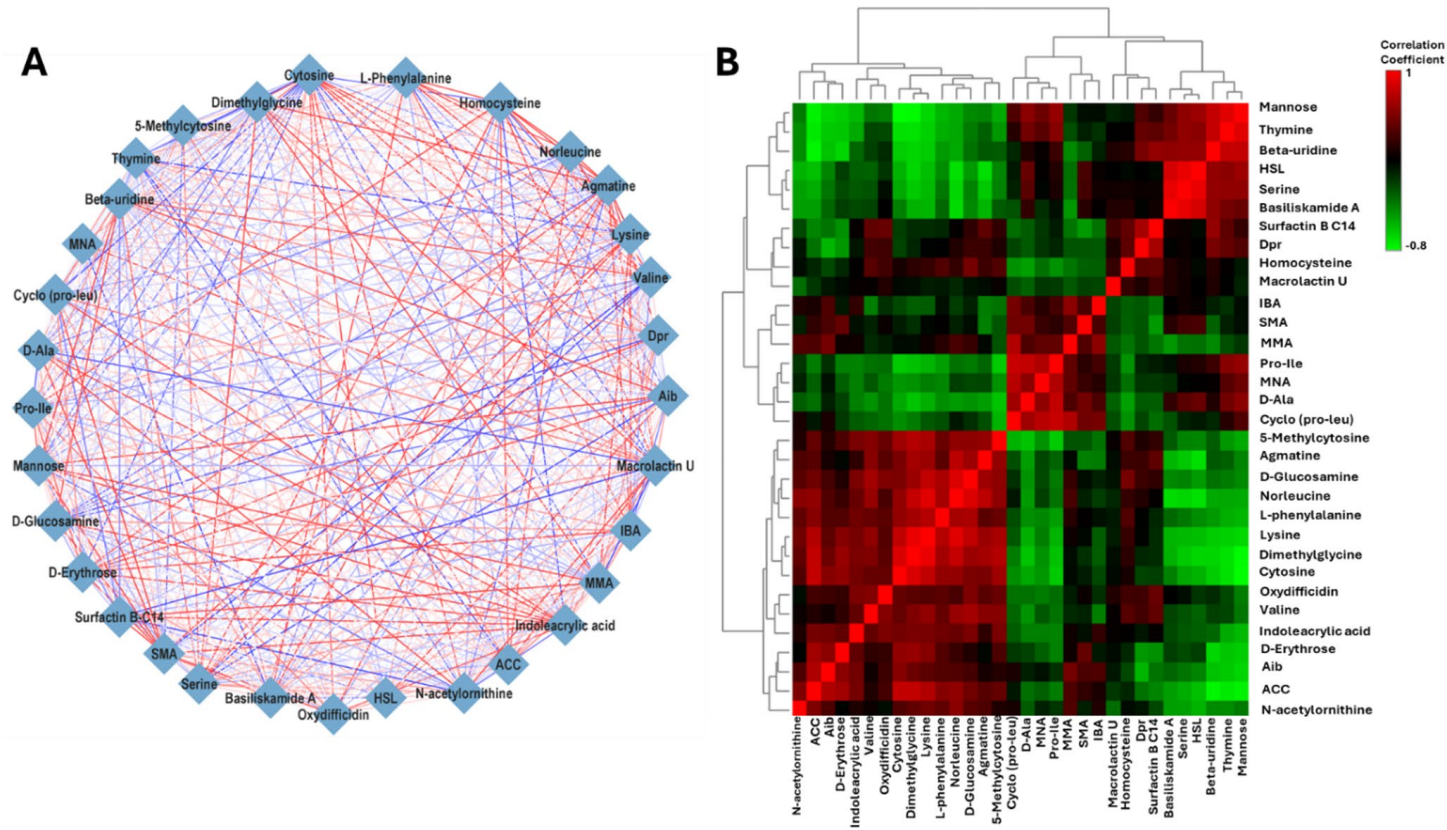
Correlation analysis of annotated metabolites from untargeted metabolomics of the endo- and exo-metabolome of *P. megaterium*. **A ** A DSPC network illustrating correlations between the top 30% of metabolites based on their *p*-values, with a 0.0005 threshold. Metabolites are depicted as nodes, and the edges are color-coded to indicate whether the correlation between connected metabolites is negative (blue) or positive (red). The thickness edges correspond to the magnitude of the correlation coefficient. **B ** A correlation heatmap generated using Spearman’s correlation coefficient for metabolite pairs. Red represents a positive correlation, while green indicates a negative correlation

**Fig. 8:**
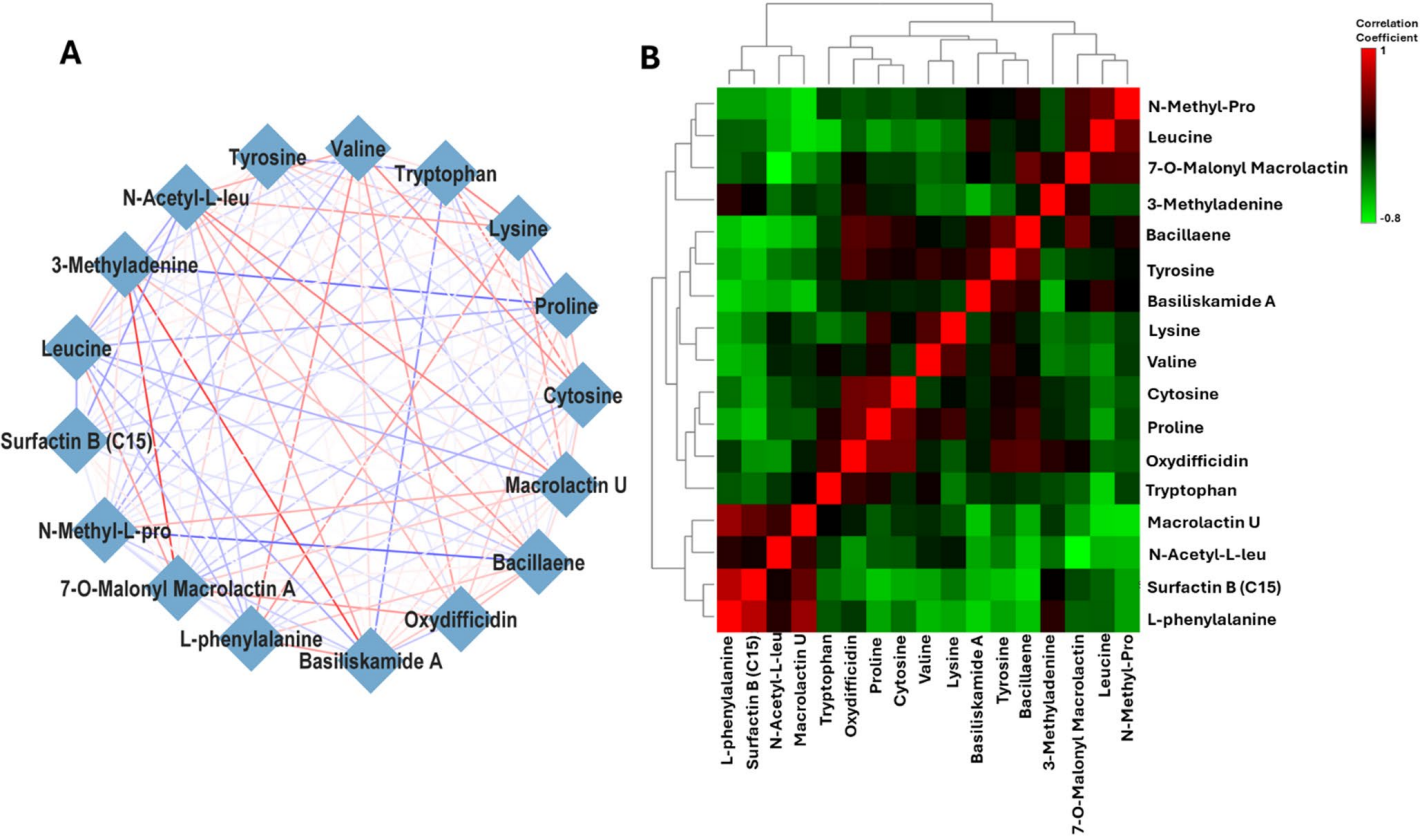
Correlation analysis of annotated metabolites from untargeted metabolomics of the endo- and exo-metabolome *B. licheniformis*. **A ** A DSPC network illustrating correlations between the top 30% of metabolites based on their *p*-values, with a 0.0005 threshold. Metabolites are depicted as nodes, and the edges are color-coded to indicate whether the correlation between connected metabolites is negative (blue) or positive (red). The edge thickness reflects the magnitude of the correlation coefficient. **B ** A correlation heatmap generated using Spearman’s correlation coefficient for metabolite pairs. Red represents a positive correlation, while green indicates a negative correlation

Additionally, correlation analysis was conducted to assess and identify overall metabolic relationships, interconnected patterns, and examine associations between endo- and exo-metabolites during co-cultivation (Figs. 7 and 8). Two approaches were employed: the Debiased Sparse Partial Correlation (DSPC) algorithm and Spearman’s correlation. DSPC operates under the assumption that the true correlations among metabolites are relatively sparse compared to the total sample size, meaning only a subset of metabolic connections are statistically significant. This method reconstructs graphical models where *p*-values and partial correlation coefficients are assigned to all metabolite pairs in the dataset, enabling the identification of metabolic connectivity with fewer samples (Basu et al. 2017). The resulting correlation networks are weighted, with each metabolite represented as a node, while edges indicate either the corresponding *p*-values or partial correlation coefficients, providing insights into metabolite interactions and regulatory influences.

The DSPC network and correlation matrix (heatmap) displayed positive correlations in red, indicating direct proportionality, and negative correlations in blue, signifying inverse proportionality, among the measured metabolites. These patterns provide insights into metabolite regulation. For instance, Fig. 8A revealed a strong positive correlation between surfactin B C14 and indole acrylic acid; L-phenyalanine and Basiliskamide A (represented by red edge), indicating that both metabolites either increased or decreased in *P. megaterium*. Conversely, there is a strong negative correlation between macrolactin U and D-alanine; as well thymine and lysine, suggesting that an increase in one metabolite coincided with a decrease in the other. A similar trend was observed in *B. licheniformis*, where a strong correlation was found between 3-methyladenine and basiliskamide A; as well as 3-methyladenine and 7-O-malonyl-macrolactin A. This suggests that these metabolites might be regulated by the same pathways, where conditions that trigger 3-methyladenine production also influence the synthesis of basiliskamide A and 7-O-malonyl-macrolactin A (Fig. 8A).

Additionally, the correlation heatmap matrix provided a more quantitative approach to data evaluation. As shown in Fig. 7B, positive correlations were observed between lipopeptides (bacilliskamide A and surfactin B) and nucleobases (beta-uridine and thymine). In contrast, strong negative correlations were found between dipeptides (proline-isoleucine and cyclo-proline-leucine) and sugars (D-erythrose and mannose), which may suggest a metabolic trade-off between peptide biosynthesis and carbohydrate metabolism. For *B. licheniformis*, positive correlations were more prevalent between amino acids (L-phenylalanine, N-acetyl-L-leucine, and Proline) and lipopeptides (Surfactin B C15, Macrolactin U, and Oxydifficidin) (Fig. 8B). This could indicate a coordinated regulation of amino acid metabolism and lipopeptide production, potentially driven by shared precursors or regulatory mechanisms.

### Summary of VOC-induced metabolic perturbations in *P. megaterium* and *B. licheniformis*

**Fig. 9:**
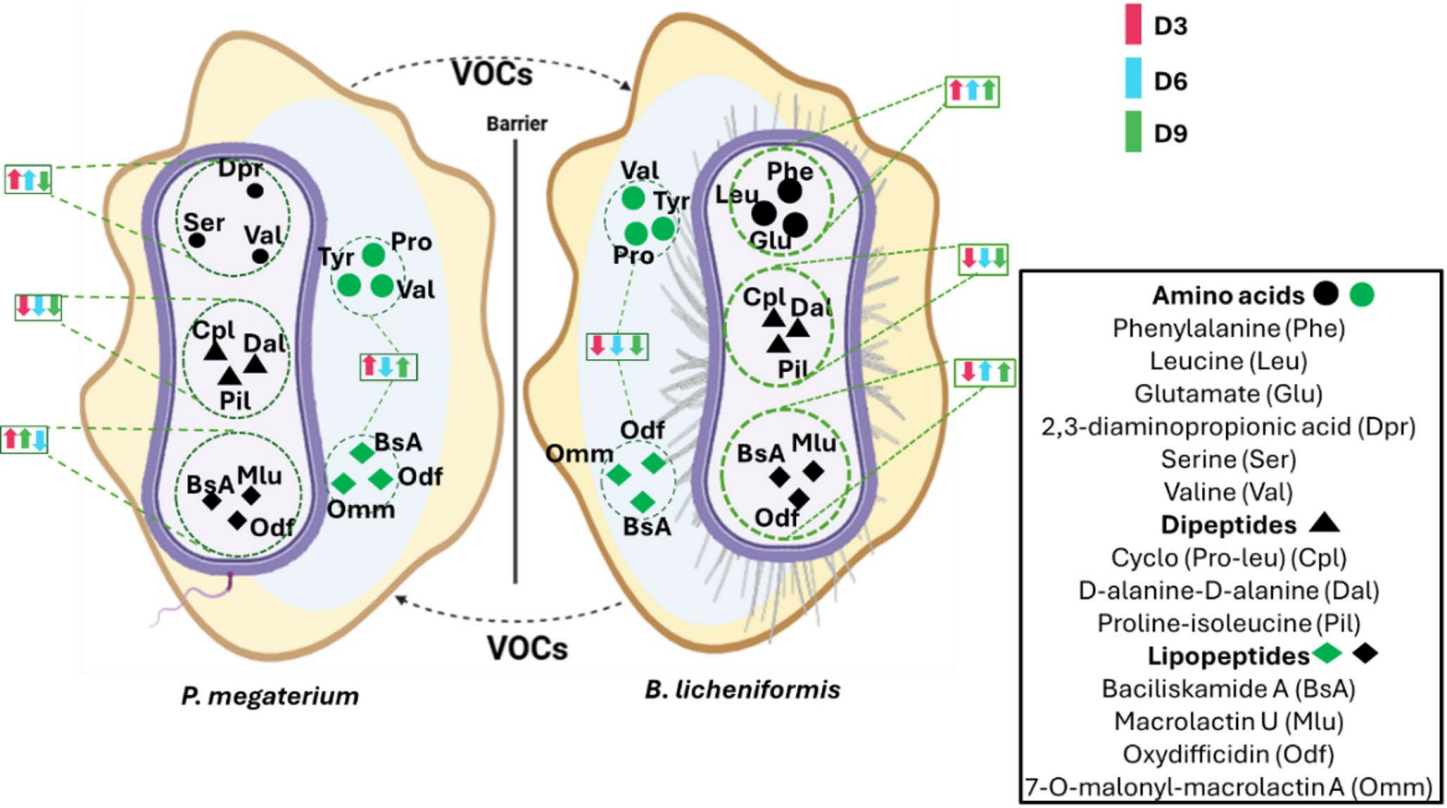
Overview of metabolic alterations induced by VOCs in both the endo- and exo-metabolomes of co-cultured bacterial strains. This figure illustrates the effects of VOCs on the intracellular (endo-) and extracellular (exo-) metabolomes of **A** *P. megaterium* and **B** *B. licheniformis*. The key metabolite classes analyzed include amino acids, dipeptides, and lipopeptides. The green dotted lines represent the endo metabolic shifts over time, and the purple dotted lines represent the exo metabolic shifts. Colored arrows indicate changes in metabolite levels: red (day 3), blue (day 6), and green (day 9), with upward arrows (↑) representing increases and downward arrows (↓) representing decreases

The overall findings are summarised in Fig. 9, which illustrates VOCs on both the intracellular (endo-) and extracellular (exo-) metabolomes of the bacterial strains studied. This figure highlights the metabolic perturbations induced by VOC exposure. For the intracellular (endo-) metabolome, amino acid levels in *P. megaterium* increased only on day 3, while in *B. licheniformis*, they increased consistently across days 3, 6, and 9. Most of the amino acids, such as proline, tyrosine, valine, phenylalanine, leucine, and glutamate, followed this pattern. This suggests that in *P. megaterium*, VOC-induced amino acid synthesis or uptake was transient, potentially due to early metabolic adjustments that later stabilised. In contrast, *B. licheniformis* maintained increased amino acid levels over time, indicating a prolonged response, possibly linked to sustained activation of biosynthetic pathways or efficient amino acid recycling.

In contrast, dipeptides showed an overall decrease in both bacterial strains, with most of the metabolites, such as cyclo-(pro-leu), d-alanine, pro-isoleucine, and glycine-leucine, being downregulated. This decrease might indicate that peptide degradation was enhanced, or that the biosynthesis of dipeptides was downregulated as cells prioritized free amino acid production over complex peptide formation in response to VOCs. Lipopeptides in *P. megaterium* exhibited an increase on days 3 and 6, while in *B. licheniformis*, they increased later, on days 6 and 9, with most of the metabolites, such as basiliskamide A, macrolactin U, and oxydifficidin, showing this trend. The early increase in *P. megaterium* lipopeptides suggests rapid changes in membrane composition to counteract VOC-induced stress. The delayed response in *B. licheniformis* might indicate a more gradual adaptation process, possibly due to differences in regulatory mechanisms governing secondary metabolism.

In the extracellular (exo-) metabolome, *P. megaterium* showed an increase in both amino acids and lipopeptides on days 3 and 9. This pattern suggests that *P. megaterium* actively released metabolites as part of its response to VOCs, potentially to influence its surrounding environment or communicate with neighboring bacteria. However, in *B. licheniformis*, these metabolite classes consistently decreased across all time points (days 3, 6, and 9), with most of the amino acids and lipopeptides, such as basiliskamide A, oxydifficidin, and 7-O-malonyl macrolactin A, being downregulated. The decrease in extracellular metabolites in *B. licheniformis* may indicate increased uptake or intracellular retention, possibly as a survival strategy to optimize internal metabolic processes under VOC-induced stress.

These results collectively indicate that VOCs play a crucial role in shaping bacterial metabolic landscapes, influencing both primary and secondary metabolism. The observed temporal variations suggest that *P. megaterium* and *B. licheniformis* respond differently to VOC exposure, with *P. megaterium* exhibiting more transient metabolic changes, while *B. licheniformis* shows more sustained intracellular shifts but reduced extracellular metabolite accumulation. This differential response may be driven by species-specific regulatory mechanisms, metabolic flexibility, or competition-driven adaptation strategies.

## Discussion

Bacterial VOCs play a crucial role in shaping microbial interactions, influencing both endo- and exo-metabolome of bacteria within communities. Bacteria do not exist in isolation but rather engage in complex inter- and intra-species interactions that are essential for survival and adaptation (Nadell et al. 2016; Yanni et al. 2019; Menezes et al. 2021). These interactions, driven by competitive and cooperative dynamics, lead to significant metabolic reprogramming compared to solitary existence (Menezes et al. 2021; Little et al. 2008). Within microbial communities, metabolites production is modulated through the exchange of signalling molecules, such as VOCs, and diffusible compounds. Certain secondary metabolites, such as antibiotics, toxins, and antifungal agents, weakly produced or absent in monoculture, but become significantly upregulated in the presence of other microbial species. This phenomenon reflects the selective activation of biosynthetic gene clusters in response to environmental cues and microbial interactions (Bertrand et al. 2014; Netzker et al. 2015). These metabolic changes affect both the intracellular (endo-metabolome) and extracellular (exo-metabolome) composition, ultimately driving microbial community dynamics and ecological functions (Garbeva et al. 2011; Tyc et al. 2017a, b). Therefore, this study employed metabolic profiling, integrating multivariate analyses, classical mass spectrometry identification, and computational tools to investigate VOC-induced alterations in the endo- and exo-metabolomes of rhizobacteria.

### Time-dependent phenotypic modifications in PGPR cultures driven by VOC exchange

PGPR often modify their phenotypic characteristics in response to environmental changes or interactions with other microorganisms. These adaptations enable them to survive and thrive under varying conditions by adjusting their metabolic and structural features (Tadrowski et al. 2018). The rapid development of filamentous structures in *B. licheniformis*, when co-cultured with *P. megaterium*, likely indicates a stress-induced or adaptive morphological response. This response may be driven by interspecies communication, possibly involving VOCs. Such morphological flexibility may support enhanced surface attachment, biofilm formation, or resource competition in mixed-species environments. Similar filamentous behaviours have been documented in *Bacillus* species facing environmental stress or microbial interactions. For instance, *B. mobilis* exhibits robust filamentous motility when interacting with metabolically inactive cells or specific environmental components such as phospholipids (Liu et al. 2020). Similarly, *B. paralicheniformis* forms organised networks with filamentous structures when colonising natural fibers (Ngom, et al. 2023). These structural transformations are often linked to metabolic rearrangements that enable bacteria to optimise survival strategies in response to environmental cues or microbial interactions. The observed filamentous structures in *B. licheniformis* suggest that VOC-mediated interactions with *P. megaterium* may have triggered a similar adaptive response. This response could potentially enhance stress resistance and resource acquisition, within the co-culture system. In contrast, the absence of filamentous structures in *P. megaterium* indicate species-specific responses to environmental cues. Different bacterial species exhibit unique morphological and metabolic adaptations based on their genetic makeup and ecological niches (Van Teeseling et al. 2017). For instance, *P. megaterium* might employ alternative strategies for coping with co-cultivation stress, such as altering its metabolic pathways or producing different types of bioactive compounds, which do not involve the formation of filamentous structures.

### Co-cultivation shifted the endo-metabolome, leading to significant metabolic adaptation

To investigate molecular perturbations induced by VOCs from neighbouring PGPR, we first analysed the metabolic variations in the endo-metabolome of the two bacterial strains. Our analysis revealed that most amino acids were more abundant in both species during co-cultivation compared to their monocultures, indicating that the influence of VOCs on amino acid metabolism. This increase was not uniform across all amino acids but was observed for specific metabolites, indicating targeted metabolic shifts rather than a global upregulation. Studies have shown that certain VOCs produced by bacteria, such as indole, 2-phenylethanol, and acetoin, have been identified as metabolic signals that can influence amino acids biosynthesis (Garbeva et al. 2014). Additionally, exposure to VOCs can trigger stress responses that lead to increased production of certain amino acids, such as glutamate and proline which play key roles in maintaining cellular homeostasis under stress (Gutiérrez-Preciado et al. 2010; Garbeva et al. 2014; Neis et al. 2015; Veselova et al. 2019). The mutual increase in amino acid production observed in both species suggests a cooperative metabolic adaptation, where VOC exchange enhances biosynthetic capacity in a way that benefits both strains.

Even though there was an increase in amino acids, the VOCs lowered the levels of dipeptides in both species. Dipeptides are not only important metabolites involved in cellular processes but are also linked to quorum sensing mechanism, which bacteria use to communicate and coordinate group behavior (Mmotla et al. 2025). Several VOCs have shown to suppress QS, influencing the regulation of peptides and other metabolites. For instance, the volatile pools of PGPR strains such as *P. fluorescence* B-4117 and *Serratia plymuthica* IC1270 inhibit QS regulation in various bacteria, including *Agrobacterium tumefaciens*, *Chromobacterium violaceum*, *Pactobacterium carotovorum*, and *P. aeruginosa*, by suppressing the transcription of acyl homoserine lactone synthase. Additionally, specific VOCs like dimethyl disulfide from *S. plymuthica* IC1270 and ketones (2-nonanone, 2-heptanone) produced by *Pseudomonas* strains have been found to disrupt bacterial quorum sensing systems (Chernin et al. 2011; Pliuta et al. 2014). This modulation of QS through VOCs suggests a cooperative adjustment, as both species shift their metabolic focus away from dipeptide-mediated signalling towards enhanced amino acid biosynthesis. Additionally, by disrupting QS, VOCs can prevent the formation of biofilms, a key factor in bacterial resistance to environmental stressors, thus helping bacteria in more dynamic or competitive environments. In the absence of biofilm formation, bacteria may be more mobile and capable of responding to environmental changes, such as nutrient availability or fluctuations in temperature and pH (Steenackers et al. 2016). This increased mobility may enable bacteria to explore new environments and search for more favourable conditions, thereby enhancing their adaptability and survival in varying ecological niches.

### Endo-metabolic adaptations of *B. licheniformis* and *P. megaterium* under VOC influence

Bacterial metabolism is a tightly controlled and dynamic process, that involves the uptake of small molecules from the environment or their synthesis from fundamental precursors (Seip and Innis 2016). To maintain stable intracellular metabolite levels, bacteria employ feedback regulation, where the production of key enzymes and transporters is modulated by final products for their respective pathways. This regulatory mechanism enables bacteria to adapt rapidly to fluctuating environmental conditions, ensuring that limited resources are allocated efficiently to support essential cellular functions as described by Seip and Innis (2016).

The most significant and affected pathway in *P. megaterium* was lysine degradation, which involves key compounds such glutaryl-CoA and 6-amino-2-oxohexanoate, along with enzyme modifications like N6-(lipoly) lysine. These intermediates were more abundant in mono-culture compared to co-culture conditions, suggesting a shift in metabolic priorities potentially driven by interactions with other bacteria. The lysine degradation pathway plays a crucial in providing building blocks for energy production and secondary metabolite synthesis. Glutaryl-CoA, a key intermediate, can be further metabolised into acetyl-CoA and succinyl-CoA, essential components of the citric acid cycle (TCA cycle). The reduction of these intermediates in co-culture could impact the TCA cycle by altering the availability of acetyl-CoA, a critical precursor for this pathway. The TCA cycle is pivotal for energy production through NADH generation. In *P. megaterium* osmotic stress has been shown to affect the TCA cycle significantly by increasing its activity to meet physiological demands. This adaptation involves redirecting metabolic fluxes towards pathways that optimize survival under stress conditions (Varela et al. 2004). Similarly, changes in the lysine degradation pathway could influence how *P. megaterium* utilises carbon sources within the TCA cycle. For instance, reduced glutaryl-CoA levels might decrease acetyl-CoA availability, potentially limiting NADH production unless compensated by other metabolic adjustments. This shift allows *P. megaterium* to redirect resources towards producing beneficial compounds such as signalling molecules.

For *B. licheniformis*, the most significant pathway identified was arginine and proline metabolism (APM), characterised by the presence of metabolites such as agmatine, 1,4-butanediamine, and L-glutamic acid. This pathway is crucial for bacterial physiology, such that L-arginine, an essential amino acid, sustains bacterial growth as both a carbon and a nitrogen source (Christgen and Becker 2019; Scribani Rossi et al. 2022). Meanwhile, L-proline synthesis typically proceeds from the central metabolite L-glutamate. These pathways are interconnected in many bacteria, including *B. subtilis*, where L-arginine and L-proline are both produced from L-glutamate. They share γ-glutamate-semialdehyde/Δ1-pyrroline-5-carboxylate as a common intermediate in their respective biosynthetic routes (Stecker et al. 2022; Scribani Rossi et al. 2022). In this study, the accumulation of precursor compounds involved in APM peaked on day 3, followed by a gradual decline over time. This temporal dynamic suggests that *B. licheniformis* initially upregulates APM, likely in response to environmental cues or nutrient availability during early growth stages. A similar trend has been observed in *B. subtilis*, where proline synthesis was triggered under osmotic stress to maintain cellular osmotic balance (Scribani Rossi et al. 2022). As conditions change over time, the reduction in these metabolites may indicate a shift towards alternative metabolic pathways or the consumption/conversion of these metabolites into other essential compounds. For instance, arginine catabolism can contribute to proline biosynthesis via ornithine, forming a metabolic shunt that enhances bacterial adaptation under fluctuating conditions. The enzyme ornithine aminotransferase facilitates this process by converting ornithine into γ-glutamate-semialdehyde/Δ-pyrroline-5-carboxylate, which subsequently leads to proline production (Scribani Rossi et al. 2022). This metabolic flexibility may enable *B. licheniformis* to efficiently manage resource utilisation and preserve cellular equilibrium. These alterations indicate that VOC sensing is closely associated with adaptive mechanisms, allowing the bacteria to respond dynamically to environmental changes.

### Interspecific differences in exo-metabolic profiles influenced by VOCs

The release of bacterial metabolites into the external environment is inevitable as detailed by Pande and Kost (2017) and the exo-metabolome encompasses all metabolites secreted outside the cell. According to the metabolic overflow theory, intracellular metabolic intermediates can be secreted when their accumulation results from imbalanced metabolic pathways (Granucci et al. 2015; Pinu et al. 2017). Metabolite secretion is considered a fundamental biochemical function that reflects the internal metabolic state of microbial cells, and it is influenced by environmental conditions. However, it has also been observed previously that some metabolites do not accumulate within the cell, they are directly secreted into the extracellular medium in response to environmental signals (Pinu et al. 2017).

In this study, VOCs played a significant role in shaping the secretion of patterns of both primary and secondary metabolites in *P. megaterium*. These VOC signals likely acted as environmental cues, influencing metabolic pathways and triggering changes in exo-metabolome composition. In co-culture *P. megaterium* exhibited a notable increase in secretion of primary metabolites. This included proline, valine, tyrosine, thymine among others and secondary metabolites such as 7-O-malonyl macrolactin A, basiliskamide A, oxydifficidin. This metabolic shift became more pronounced on day 9, indicating that prolonged exposure to VOCs enhances biosynthetic activity overtime. The regulation of metabolite secretion in response to VOCs aligns with the metabolic overflow theory (Pinu et al. 2017). However, the strong positive correlation observed between amino acids and lipopeptides suggests that some metabolites may be regulated by shared biosynthetic pathways rather than simple metabolic overflow.

In *B. licheniformis*, the secretion of both primary and secondary metabolites was notably reduced under co-culture conditions, with the most significant decline on 9. The observed decrease in metabolite secretion may indicate metabolic regulatory suppression. This suggest that *B. licheniformis* adjusted its biosynthetic pathways in response to neighbouring strain. As previously discussed, VOC-induced metabolic flexibility allows bacteria to optimise resource utilization and maintain cellular homeostasis (Pinu et al. 2017). Therefore, *B. licheniformis* may have reallocated intracellular metabolites to essential cellular functions instead of excreting them. This could serve as a survival strategy in shared environment. Moreover, the correlation matrix revealed positive associations between several compounds. These included amino acids (L-phenylalanine, N-acetyl-L-leucine, and proline) and lipopeptides, fatty alcohol and phospholipid (Surfactin B C15, Macrolactin U, and Oxydifficidin). These findings support the idea that co-cultivation alters biosynthetic priorities. This interspecific metabolic coordination suggests a form of positive cooperation. In this scenario, the metabolic adjustments of one strain may create a more favourable biochemical environment for the other. On day 9, the secretion of secondary metabolites, such as Bacilliskamide A and 7-O-malonyl macrolactin A, was reduced. This suggests that their production may be tightly regulated by environmental signals. This regulation may involve cross-regulation between amino acid metabolism and secondary metabolite biosynthesis. These findings align with previous studies that emphasise the role of environmental cues in modulating secondary metabolite production in *Bacillus* species. For instance, *P. megaterium* has shown that its metabolite production is highly responsive to external conditions. These conditions include nutrient availability and microbial interactions. Such factors influence the synthesis of bioactive compounds such as fatty acids and hydrocarbons (Hur et al. 2024). The ability of *P. megaterium* to enhance metabolite secretion while *B. licheniformis* downregulates certain pathways highlights the species-specific metabolic plasticity. This plasticity appears to be driven by VOCs (Shen and Chou 2016). However, this is not merely a one-sided effect. The observed shifts suggest a form of positive cooperativity between the two species. In this case, VOC-induced metabolic changes in one species create conditions that facilitate biosynthetic adjustment in the other. For example, *P. megaterium* enhances metabolite secretion in response to VOCs. Meanwhile, *B. licheniformis* appears to regulate its metabolic pathways to maintain intracellular balance. This dynamic suggests that interspecies VOC signaling does not simply alter metabolism in isolation but promotes a coordinated metabolic adjustment that optimizes overall biosynthetic efficiency in both species.

## Conclusion

The findings of this study highlight the crucial role bacterial VOCs play in shaping microbial interactions and driving metabolic reprogramming within bacterial communities. VOCs induce significant changes in both the endo-and exo-metabolomes of bacteria, prompting selective changes in their metabolic processes. These VOC -mediated interactions between *P. megaterium* and *B. licheniformis* revealed distinct species-specific metabolic adaptations, suggesting that the presence of one species can dramatically influence the metabolic behavior of the other. Notably, morphological changes such as filamentous growth in *B. licheniformis* suggest that VOC-mediated signalling enhances microbial adaptation strategies. Furthermore, these interactions led to species-specific metabolic adaptations, with *P. megaterium* exhibiting increased metabolite secretion, including amino acids and secondary metabolites such as macrolactins and baciliskamide A, while *B. licheniformis* retained intracellular metabolites, likely as a survival strategy. These shifts reflect the differential regulation of metabolic pathways, such as lysine degradation and arginine-proline metabolism, and collectively point to VOC-induced metabolic plasticity as a critical mechanism for microbial adaptation in dynamic environments like rhizosphere.

Beyond mechanistic insights, this study provides a framework for leveraging VOC-based microbial communication to enhance sustainable agriculture. By selecting or engineering compatible PGPR capable of cooperative interactions via VOCs, it may be possible to reduce reliance on synthetic fertilizers and pesticides while improving crop health and productivity. Such microbial consortia could foster more resilient plant-microbe ecosystems that support soil health and ecological balance. However, this study is limited by its laboratory-scale design and focus on only two bacterial species. Future work should incorporate more complex community settings, explore plant responses to these VOC-mediated interactions, and validate findings in soil or field environments. Integrating transcriptomics and proteomic data may also help elucidate regulatory mechanisms underlying VOC-driven metabolic responses. Overall, this research contributes to a growing body of evidence that VOCs are central to microbial communication and adaptation, with promising implications for microbial biotechnology and agriculture, ultimately laying the groundwork for development of next-generation PGPR formulations that harness VOC-mediated synergy to enhance crop resilience and crop optimize soil microbiome function.

## Supplementary Information

Below is the link to the electronic supplementary material.

Supplementary Material 1

## Data Availability

No datasets were generated or analysed during the current study.
