# Supplementary material for: Metabolomic profiling of VOC-driven interactions between Priestia megaterium and Bacillus licheniformis in a simulated rhizosphere using split petri dishes
Source: Arch Microbiol. 2025 Aug 12;207(9):224. doi: 10.1007/s00203-025-04426-9 (PMC12343639; doi:10.1007/s00203-025-04426-9)
Supplement: Supplementary file 1 — Supplementary Material 1 [file 203_2025_4426_MOESM1_ESM.docx]

**Kamogelo Mmotla^a^, Farhahna Allie^a^, Thendo Mafuna^a^, Manamele D Mashabela^a,b*^ and Msizi I Mhlongo^a,b*^**

^a^Department of Biochemistry, Faculty of Science, University of Johannesburg, Auckland Park, 2006, South Africa.

^b^Research Centre for Plant Metabolomics, Faculty of Science, University of Johannesburg, Auckland Park, 2006, South Africa
*Correspondence: University of Johannesburg, South Africa. E-mail: [manamelem@uj.ac.za](mailto:manamelem@uj.ac.za); +27-11-559-4573 (M.D.M); [mmhlongo@uj.ac.za](mailto:mmhlongo@uj.ac.za); Tel.: +27-11-559-4573 (M.M)

**Supplementary files**

**
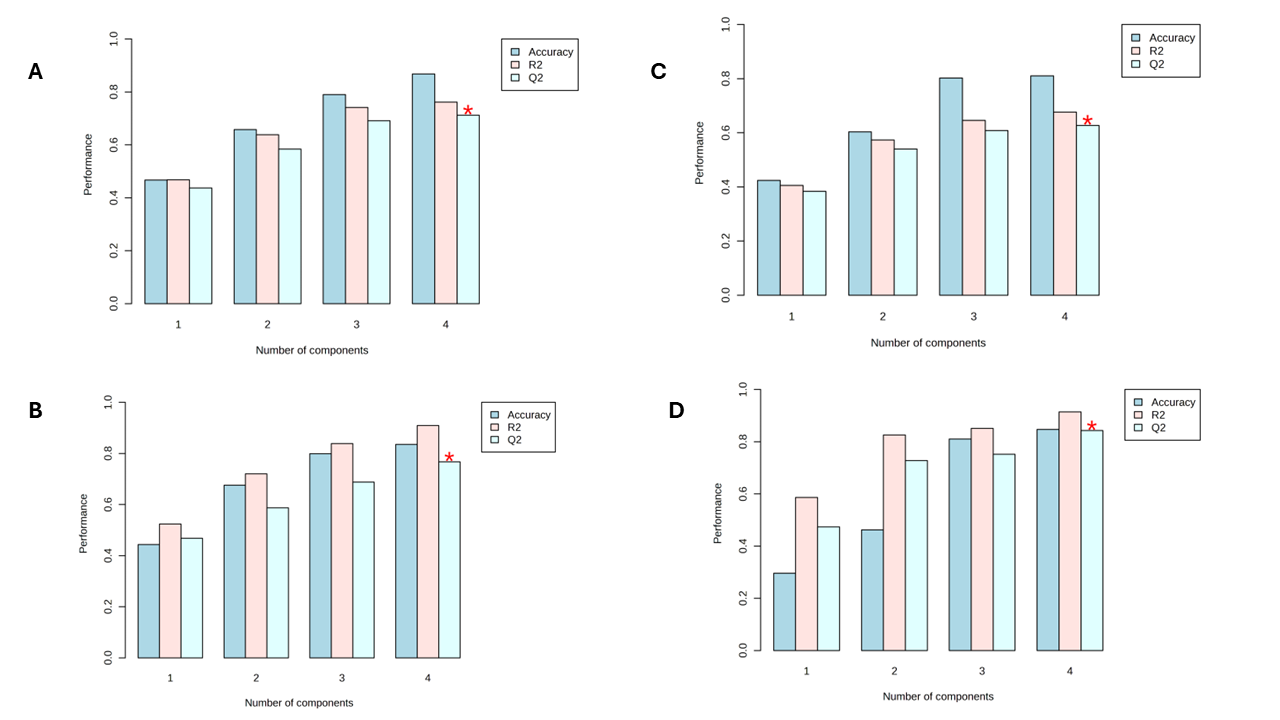
**

**Figure S1.1: The cross-validated performance of the PLS-DA model across four different datasets.** *P. megaterium* endometabolome **(A)**, exometabolome **(B),** and *B. licheniformis* endometabolome **(C)**, exometabolome **(D)**. For each dataset, bar plots display three key performance metrics: Classification Accuracy (light blue), R2 (pink), and Q2 (light cyan), as a function of the number of components (1-4). For each dataset, the red asterisk identifies the component count that maximizes Q2 while maintaining a small R2-Q2 gap, thereby designating the model with the best cross-validtion performance and generalization ability.

**
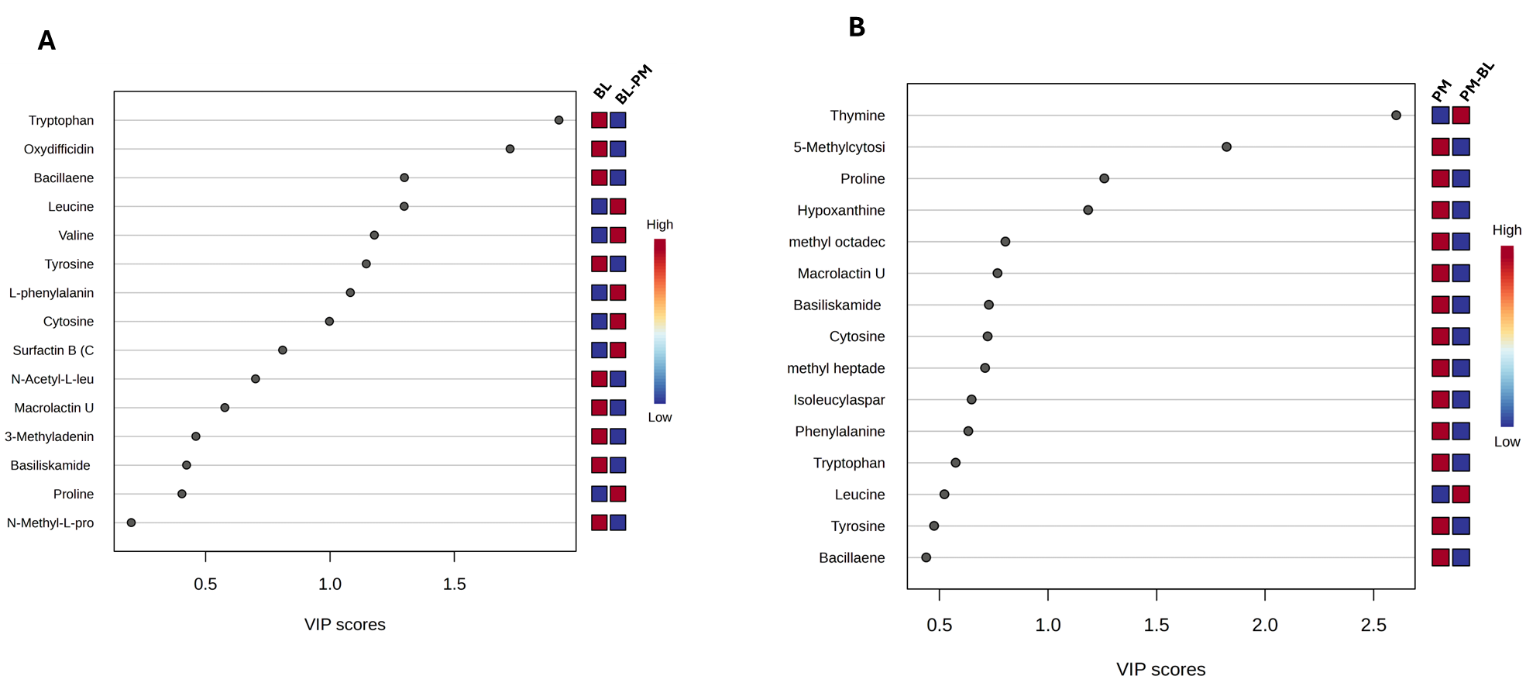
**

**Figure S1.2: Variable Importance in Projection (VIP) scores from PLS-DA models for *B. licheniformis*** **(BL) and *P. megaterium* (PM)**. Each plot displays metabolites ranked by VIP score, with a heatmap besides each indicating their relative abundance across the groups compared between monoculture and co-culture.

**Table S1.1:** A summary of the annotated and putatively identified metabolites from monoculture and co-culture samples of *P. megaterium*. A tick mark (✔) signifies the presence of a metabolite in a specific sample, either in the endo- or exo-metabolome while a dash (**X**) signifies it was not identified. The qualitative and quantitative distribution is depicted in the heatmaps (Figure 1.2 and 1.5).

| **No** | **Compound name** | **Formula** | **rt (min)** | **m/z** | **Fragments** | **Adduct** | **Endo-metabolme** | **Exo-metabolome** |
| --- | --- | --- | --- | --- | --- | --- | --- | --- |
| 1 | N-Acetyl-L-leucine | C8H15NO3 | 1.22 | 172.09 | 130 | [M+H]+ | **X** | **✔** |
| 2 | beta-uridine | C9H12N2O6 | 1.337 | 245.08 | 142 | [M+H]+ | **✔** | **X** |
| 3 | 1-Imidazoleacetic acid | C5H6N2O2 | 1.463 | 127.0499 | 42,69,109 | [M+H]+ | **✔** | **X** |
| 4 | Homocysteine | C4H9NO2S | 1.604 | 136.0414 | 56,90.115 | [M+H]+ | **✔** | **X** |
| 5 | 5-Methylcytosine | C5H7N3O | 1.668 | 126.0605 | 83, 109,114 | [M+H]+ | **✔** | **X** |
| 6 | L-Phenylalanine | C9H11NO2 | 2.141 | 166.0873 | 120,153 | [M+H]+ | **✔** | **✔** |
| 7 | Indoleacrylic acid | C11H9NO2 | 2.21 | 188.07 | 170 | [M+H]+ | **✔** | **X** |
| 8 | 3-Methyadenine | C6H7N5 | 2.821 | 150.0908 | 112,141 | [M+H]+ | **✔** | **X** |
| 9 | 1-Aminocyclopropane-1-carboxylic acid | C4H7NO2 | 3.017 | 102.0616 | 56 | [M+H]+ | **✔** | **X** |
| 10 | Citramalate | C5H8O5 | 3.089 | 149.0439 | 113,133 | [M+H]+ |  | **✔** |
| 11 | Fumaric acid | C4H4O4 | 3.091 | 117.0088 | 113,89 | [M+H]+ |  | **✔** |
| 12 | 1,2-Cyclohexanedione | C6H8O2 | 3.237 | 113.064 | 70,100 | [M+H]+ | **✔** | **X** |
| 13 | Agmatine | C5H14N4 | 3.325 | 131.1399 | 72, 100 | [M+H]+ | **✔** | **X** |
| 14 | 3-Methyl-2-Oxindole | C9H9NO | 3.44 | 148.08 | 130,149 | [M+H]+ | **✔** | **✔** |
| 15 | N-acetylornithine | C7H14N2O3 | 3.49 | 175.11 | 115,133,157,158 | [M+H]+ | **✔** | **X** |
| 16 | Mannose | C6H12O6 | 3.51 | 203.05 | 129,202 | [M+H]+ | **✔** | **X** |
| 17 | Acetyl Arginine | C8H16N4O3 | 3.53 | 217.13 | 112,113,115,139,157 | [M+H]+ | **✔** | **X** |
| 18 | Tyrosine | C9H11NO3 | 3.71 | 182.11 | 119,123,136,147,165 | [M+H]+ |  | **✔** |
| 19 | Basiliskamide A | C23H31NO4 | 4.89 | 386.2113 | 216, 149 | [M+H]+ | **✔** | **✔** |
| 20 | Thymine | C5H6N2O2 | 4.893 | 127.0455 | 44,56,71 | [M+H]+ | **✔** | **✔** |
| 21 | 7-O-Malonyl macrolactin A | C24H35O5 | 4.95 | 511.2463 | 425,349,367 | [M+Na]+ | **✔** | **✔** |
| 22 | 4-Hydroxy-6-Methylpyran-2-One | C6H6O3 | 5.18 | 127.06 | 108,109 | [M+H]+ | **X** | **✔** |
| 23 | Indole-3-ethanol | C10H11NO | 5.31 | 144.08 | 100,116,117 | [M+H]+ | **✔** | **✔** |
| 24 | Cytosine | C4H5N3O | 5.314 | 112.0563 | 69, 102 | [M+H]+ | **✔** | **✔** |
| 25 | N-Methylnicotinamide | C7H8N2O | 5.531 | 137.0737 | 80,100 | [M+H]+ | **✔** | **X** |
| 26 | Macrolactin U | C24H35O5 | 5.88 | 503.0865 | 243, 217 | [M+Na]+ | **✔** | **✔** |
| 27 | D-Erythrose | C4H8O4 | 6.005 | 121.0513 | 77 | [M+H]+ | **✔** | **✔** |
| 28 | N6-Acetyl-L-lysine | C8H16N2O3 | 6.184 | 189.1307 | 56, 126,147 | [M+H]+ | **✔** | **X** |
| 29 | 6,10-dimethyl-5,9-undecadien-2-one | C13H22O | 6.235 | 217.1584 | 136,253 | [M+Na]+ |  | **✔** |
| 30 | Decanoyl-L-carnitine | C17H33NO4 | 6.29 | 316.23 | 257 | [M+H]+ | **✔** | **X** |
| 31 | N-Acetyl-L-Phenylalanine | C11H13NO3 | 6.33 | 208.11 | 120,121,131,149,162 | [M+H]+ | **✔** |  |
| 32 | 2-Phenylacetamide | C8H9NO | 6.517 | 136.0825 | 44,91,111 | [M+H]+ | **✔** | **✔** |
| 33 | Indole-3-acetyl-L-leucine | C16H20N2O3 | 6.91 | 289.15 | 130,132,243,271 | [M+H]+ | **✔** | **X** |
| 34 | 4-Hydroxy-4-methyl-2-pentanone | C6H12O2 | 7.052 | 117.0765 | 47,75 | [M+H]+ | **X** | **✔** |
| 35 | 6-methylpiperidine-2-carboxylic acid | C7H13NO2 | 7.075 | 144.085 | 81,116 | [M+H]+ | **X** | **✔** |
| 36 | Indole-3-butanoic acid | C12H13NO2 | 7.09 | 204.1 | 130,144,186 | [M+H]+ | **✔** | **X** |
| 37 | Indolbutyric acid | C12H13NO2 | 7.1 | 186.11 | 144,158 | [M+H]+ |  | **✔** |
| 38 | Valine | C5H11NO2 | 7.159 | 118.0894 | 55,72 | [M+H]+ | **✔** | **✔** |
| 39 | piperazine-2,5-dione | C4H6N2O2 | 7.185 | 137.0325 | 120,137 | [M+Na]+ | **✔** | **X** |
| 40 | Hypoxanthine | C5H4N4O | 7.191 | 137.0417 | 120 | [M+H]+ | **X** | **✔** |
| 41 | Dimethylglycine | C4H9NO3 | 7.192 | 104.0774 | 44,58 | [M+H]+ | **✔** | **X** |
| 42 | Cyclo(proline-leucine) | C11H18N2O2 | 7.45 | 211.12 | 127,183 | [M+H]+ | **✔** | **X** |
| 43 | Lysine | C6H14N2O2 | 7.742 | 147.0986 | 84,118 | [M+H]+ | **✔** | **✔** |
| 44 | D-(+)-Glucosamine | C6H13NO5\| | 7.823 | 180.09236\| | 116,162 | [M+H]+ | **✔** | **X** |
| 45 | Isoleucylaspartate | C10H18N2O5 | 8.002 | 247.1403 | 86 | [M+H]+ | **X** | **✔** |
| 46 | Oxydifficidin | C31H45O7P | 8.26 | 561.0193 | 560,545 | [M+H]+ | **✔** | **✔** |
| 47 | Norleucine | C6H13NO2 | 8.393 | 132.113 | 69,86,120 | [M+H]+ | **✔** | **X** |
| 48 | Homoserine lactone | C4H7NO2 | 9.11 | 312.26 | 211,284,294,308 | [M+H]+ | **✔** | **X** |
| 49 | Leucine | C6H13NO2 | 9.394 | 132.0996 | 62,86 | [M+H]+ | **X** | **✔** |
| 50 | N-Methylcytisine | C12H16N2O | 9.558 | 227.1078 | 116,205 | [M+Na]+ | **✔** | **✔** |
| 51 | Serine | C3H7NO3 | 9.846 | 106.0606 | 60,70,88 | [M+H]+ | **✔** | **X** |
| 52 | Proline | C5H9NO2 | 10.002 | 116.0678 | 70 | [M+H]+ | **X** | **✔** |
| 53 | D-Ala-D-ala | C6H12N2O3 | 10.045 | 161.0833 | 90,120 | [M+H]+ | **✔** | **X** |
| 54 | 2-amino-2-methylpropanoate | C4H9NO2 | 10.358 | 104.0655 | 58 | [M+H]+ | **✔** | **X** |
| 55 | Pyridoxamine | C8H12N2O2 | 10.702 | 169.1031 | 116,152 | [M+H]+ | **✔** | **X** |
| 56 | 1-Acetylimidazole | C5H6N2O | 10.711 | 111.0573 | 56,69,83 | [M+H]+ | **✔** | **X** |
| 57 | 7-O-Succinyl macrolactin A | C28H38O8 | 11.45 | 525.34 | 385, 367,349 | [M+Na]+ | **✔** | **X** |
| 58 | L-Prolyl-L-isoleucine | C11H20N2O3 | 11.5 | 229.15 | 183,230 | [M+H]+ | **✔** | **X** |
| 59 | Acetylcholine | C7H16NO2 | 11.794 | 147.1366 | 60,124 | [M+H]+ | **✔** | **X** |
| 60 | Surfactin B-C14 | C51H91O14N7 | 13.03 | 1026.629 | 1008, 664, 452 | [M+H]+ | **✔** | **X** |
| 61 | L-2,3-diaminopropionic acid | C3H8N2O2 | 13.126 | 105.0847 | 59,76,88 | [M+H]+ | **✔** | **X** |
| 62 | methyl octadecanoate | C19H38O2 | 13.31 | 299.3 | 257,280 | [M+H]+ | **X** | **✔** |
| 63 | methyl heptadecanoate | C18H36O2 | 13.81 | 285.29 | 117 | [M+H]+ | **X** | **✔** |
| 64 | Surfactin C | C53H93N7O13 | 14.01 | 1036.698 | 685, 1018 | [M+H]+ | **X** | **✔** |
| 65 | Tryptophan | C11H12N2O2 | 15.11 | 205.12 | 146,159,170 | [M+H]+ | **X** | **✔** |
| 66 | Surfactin B (C14) | C51H91O14N7 | 15.77 | 1026.804 | 542, 672 | [M+H]+ | **X** | **✔** |

**Table S4.2:** A summary of the annotated and putatively identified metabolites from monoculture and co-culture samples of *B. licheniformis*. A tick mark (✔) signifies the presence of a metabolite in a specific sample, either in the endo- or exo-metabolome while a dash (**X**) signifies it was not identified. The qualitative and quantitative distribution is depicted in the heatmaps (Figure 1.2 and 1.5).

| **No** | **Compound name** | **Formula** | **rt (min)** | **m/z** | **Fragments** | **Adduct** | **Endo-metabolome** | **Exo-metabolome** |
| --- | --- | --- | --- | --- | --- | --- | --- | --- |
| 1 | Indole-3-carbinol | C9H9NO | 0.878 | 130.0679 | 102,103,57 | [M+H-H2O]+ **X** | | ✔ |
| 2 | 1,4-butanediamine | C10H26N4 | 0.896 | 203.2312 | 112,162 | [M+H]+ | ✔ | **X** |
| 3 | 4-Fluoraniline | C6H6FN | 0.993 | 112.0646 | 103,92 | [M+H]+ | **X** | ✔ |
| 4 | D-Glucose | C6H12O6 | 1.07 | 383.13 | 204206 | [M+H]+ | ✔ | **X** |
| 5 | Tryptophan | C11H12N2O2 | 1.1 | 205.12 |  | [M+H]+ | **X** | ✔ |
| 6 | N-Acetyl-L-leucine | C8H15NO3 | 1.22 | 172.09 | 130 | [M+H]+ | **X** | ✔ |
| 7 | Acetyl-L-lysine | C8H16N2O3 | 1.42 | 189.13 | 1.26E+11 | [M+H]+ | ✔ | **X** |
| 8 | 2-Phenylacetamide | C8H9NO | 1.541 | 136.0779 | 118,44,77 | [M+H]+ | ✔ | ✔ |
| 9 | 5-Methylcytosine | C5H7N3O | 1.662 | 126.0605 | 83109116 | [M+H]+ | ✔ | ✔ |
| 10 | 1-Aminocyclopropane-1-carboxylic acid | C4H7NO2 | 1.894 | 102.0576 | 56 | [M+H]+ | ✔ | ✔ |
| 11 | L-Glutamic acid | C5H9NO4 | 1.894 | 148.0626 | 130186 | [M+H]+ | ✔ | **X** |
| 12 | Indole-3-propionic acid | C11H11NO2 | 2.23 | 190.09 | 130172 | [M+H]+ | ✔ | **X** |
| 13 | Creatinine | C4H7N3O | 2.234 | 114.072 | 44,42 | [M+H]+ | **X** | ✔ |
| 14 | Valine | C5H11NO2 | 2.524 | 118.0937 | 55,72 | [M+H]+ | ✔ | ✔ |
| 15 | Adenine | C5H5N5 | 2.604 | 136.0642 | 100137 | [M+H]+ | ✔ | **X** |
| 16 | 3-METHYLADENINE | C6H7N5 | 2.814 | 150.086 | 129146 | [M+H]+ | ✔ | ✔ |
| 17 | Fumaric acid | C4H4O4 | 3.085 | 117.0088 | 114,1143,88 | [M+H]+ | **X** | ✔ |
| 18 | Citramalate | C5H8O5 | 3.111 | 149.0487 | 133 | [M+H]+ | ✔ | **X** |
| 19 | L-Phenylalanine | C9H11NO2 | 3.139 | 166.0773 | 101120 | [M+H]+ | ✔ | ✔ |
| 20 | Arginine butyl ester | C10H22N4O2 | 3.31 | 231.17 | 172214 | [M+H]+ | ✔ | **X** |
| 21 | Agmatine | C5H14N4 | 3.328 | 131.1355 | 72 | [M+H]+ | ✔ | **X** |
| 22 | 3-Methyl-2-oxindole | C9H9NO | 3.47 | 148.07 | 130133 | [M+H]+ | ✔ | **X** |
| 23 | 2-Heptylquinoline-3,4-diol | C16H21NO2 | 3.58 | 288.2 | 175 | [M+H]+ | ✔ | **X** |
| 24 | Acetyl arginine | C8H16N4O3 | 3.61 | 217.13 | 112, 113, 115, 139, 159, 199 | [M+H]+ | ✔ | **X** |
| 25 | N-acetylornithine | C7H14N2O3 | 3.64 | 175.11 | 112, 115, 157,158 | [M+H]+ | ✔ | **X** |
| 26 | 2-Aminobiphenyl | C12H11N | 3.7 | 170.1 | 128152 | [M+H]+ | ✔ | **X** |
| 27 | Indole-3-Ethanol | C10H11NO | 3.75 | 162.09 | 1.44E+08 | [M+H]+ | ✔ | **X** |
| 28 | N-Tigloylglycine | C7H11NO3 | 4.546 | 158.091 | 83116 | [M+H]+ | ✔ | **X** |
| 29 | Basiliskamide A | C23H31NO4 | 4.89 | 386.2113 | 216, 149 | [M+H]+ | ✔ | ✔ |
| 30 | N-Acetyl-ornithine | C7H14N2O3 | 5.221 | 175.0972 | 43115 | [M+H]+ | ✔ | **X** |
| 31 | Cytosine | C4H5N3O | 5.291 | 112.0563 | 69 | [M+H]+ | ✔ | **X** |
| 32 | Indolbutyric acid | C12H13NO2 | 5.39 | 186.09 | 144, 168 | [M+H]+ | ✔ | **X** |
| 33 | Leucine | C6H13NO2 | 5.701 | 132.1085 | 62,86 | [M+H]+ | ✔ | ✔ |
| 34 | N-Methyl-L-proline | C6H11NO2 | 5.705 | 130.0812 | 107,84 | [M+H]+ | **X** | ✔ |
| 35 | Macrolactin U | C31H44O4 | 5.89 | 503.13 | 230 | [M+Na]+ |  | ✔ |
| 36 | D-pantothenic acid | C9H17NO5 | 5.95 | 220.12 | 116, 124, 142, 160, 174, 184 | [M+H]+ | ✔ | **X** |
| 37 | Prolylphenylalanine | C14H18N2O3 | 6.492 | 263.1402 | 202,116,84 | [M+H]+ | **X** | ✔ |
| 38 | 3-aminoisobutanoate | C4H9NO2 | 6.594 | 104.0655 | 86,57 | [M+H]+ | **X** | ✔ |
| 39 | 4-O-Methylphloracetophenone | C9H10O4 | 6.63 | 183.06 | 141, 165,182 | [M+H]+ | ✔ | **X** |
| 40 | Niacinamide | C6H6N2O | 6.835 | 123.0506 | 80,96,102 | [M+H]+ | ✔ | **X** |
| 41 | N-Aceyl-L-tyrosine | C11H13NO4 | 6.86 | 224.09 | 136, 178,182,206 | [M+H]+ | ✔ | **X** |
| 42 | 6-methylpiperidine-2-carboxylic acid | C7H13NO2 | 7.057 | 144.099 | 116107 | [M+H]+ | **X** | ✔ |
| 43 | 4-Hydroxy-4-methyl-2-pentanone | C6H12O2 | 7.062 | 117.0722 | 75,43 | [M+H]+ | **X** | ✔ |
| 44 | Hypoxanthine | C5H4N4O | 7.188 | 137.0417 | 124138 | [M+H]+ | **X** | ✔ |
| 45 | 6,10-dimethyl-5,9-undecadien-2-one | C13H22O | 7.648 | 217.1526 | 194253 | [M+Na]+ | **X** | ✔ |
| 46 | Lysine | C6H14N2O2 | 7.743 | 147.0986 | 84,129, | [M+H]+ | ✔ | ✔ |
| 47 | 2-Amino-2-Methylpropanoate | C4H9NO2 | 7.766 | 104.0615 | 58 | [M+H]+ | ✔ | **X** |
| 48 | Oxydifficidin | C31H45O7P | 8.26 | 561.0193 | 560545 | [M+H]+ | ✔ | ✔ |
| 49 | Bacillaene | C34H48N2O6 | 8.65 | 581.359 | 225432 | [M+H]+ | ✔ | ✔ |
| 50 | 7-O-Malonyl macrolactin A | C27H36O8 | 8.68 | 511.378 | 425349 | [M+Na]+ | **X** | ✔ |
| 51 | Gly-Leu | C8H16N2O3 | 9.77 | 189.12 | 1.32E+08 | [M+H]+ | ✔ | **X** |
| 52 | D-Ala-D-ala | C6H12N2O3 | 10.05 | 161.0882 | 90119 | [M+H]+ | ✔ | **X** |
| 53 | Arginine | C6H14N4O2 | 10.138 | 175.123 | 116 | [M+H]+ | ✔ | **X** |
| 54 | Arginine ethyl ester | C8H18N4O2 | 10.17 | 203.15 | 144186 | [M+H]+ | ✔ | **X** |
| 55 | L-Prolyl-L-isoleucine | C11H20N2O3 | 10.73 | 229.15 | 183 | [M+H]+ | ✔ | **X** |
| 56 | Thymine | C5H6N2O2 | 12.282 | 127.0499 | 44,71,81 | [M+H]+ | ✔ | **X** |
| 57 | L-2,3-Diaminopropanoic acid | C3H8N2O2 | 13.126 | 105.0807 | 59,76 | [M+H]+ | ✔ | **X** |
| 58 | Surfactin B-C14 | C51H91O14N7 | 13.56 | 1026.629 | 664452 | [M+H]+ | ✔ | **X** |
| 59 | Methyl heptadecanoate | C18H36O2 | 13.63 | 285.28 | 103 | [M+H]+ | **X** | ✔ |
| 60 | D-Erythrose | C4H8O4 | 13.924 | 121.0427 | 103,93 | [M+H]+ | **X** | ✔ |
| 61 | Surfactin A-C15 | C53H93O13N7 | 14.44 | 1036.61 | 1008, 790 | [M+H]+ | ✔ | ✔ |
